# Assessing the multi-scale predictive ability of ecosystem functional attributes for species distribution modelling

**DOI:** 10.1371/journal.pone.0199292

**Published:** 2018-06-18

**Authors:** Salvador Arenas-Castro, João Gonçalves, Paulo Alves, Domingo Alcaraz-Segura, João P. Honrado

**Affiliations:** 1 Centro de Investigação em Biodiversidade e Recursos Genéticos (InBIO/CIBIO-ICETA), Universidade do Porto, Vairão, Portugal; 2 Departamento de Botánica, Facultad de Ciencias, Universidad de Granada, Granada, Spain; 3 Andalusian Center for the Assessment and Monitoring of Global Change (CAESCG), Universidad de Almería, Almería, Spain; 4 iecolab. Interuniversitary Institute for Earth System Research (IISTA), Universidad de Granada, Granada, Spain; 5 Faculdade de Ciências, Universidade do Porto, Porto, Portugal; Kerala Forest Research Institute, INDIA

## Abstract

Global environmental changes are rapidly affecting species’ distributions and habitat suitability worldwide, requiring a continuous update of biodiversity status to support effective decisions on conservation policy and management. In this regard, satellite-derived Ecosystem Functional Attributes (EFAs) offer a more integrative and quicker evaluation of ecosystem responses to environmental drivers and changes than climate and structural or compositional landscape attributes. Thus, EFAs may hold advantages as predictors in Species Distribution Models (SDMs) and for implementing multi-scale species monitoring programs. Here we describe a modelling framework to assess the predictive ability of EFAs as Essential Biodiversity Variables (EBVs) against traditional datasets (climate, land-cover) at several scales. We test the framework with a multi-scale assessment of habitat suitability for two plant species of conservation concern, both protected under the EU Habitats Directive, differing in terms of life history, range and distribution pattern (*Iris boissieri* and *Taxus baccata*). We fitted four sets of SDMs for the two test species, calibrated with: interpolated climate variables; landscape variables; EFAs; and a combination of climate and landscape variables. EFA-based models performed very well at the several scales (AUC_median_ from 0.881±0.072 to 0.983±0.125), and similarly to traditional climate-based models, individually or in combination with land-cover predictors (AUC_median_ from 0.882±0.059 to 0.995±0.083). Moreover, EFA-based models identified additional suitable areas and provided valuable information on functional features of habitat suitability for both test species (narrowly *vs*. widely distributed), for both coarse and fine scales. Our results suggest a relatively small scale-dependence of the predictive ability of satellite-derived EFAs, supporting their use as meaningful EBVs in SDMs from regional and broader scales to more local and finer scales. Since the evaluation of species’ conservation status and habitat quality should as far as possible be performed based on scalable indicators linking to meaningful processes, our framework may guide conservation managers in decision-making related to biodiversity monitoring and reporting schemes.

## Introduction

Global environmental changes are affecting species distributions and ecosystem functioning worldwide, with profound effects in terms of loss and relocation of biodiversity [1,2]. Thus, a continuous update of biodiversity status and the effectiveness of conservation policies are international goals for the coming years [3,4]. Different approaches combining statistical modelling tools and biodiversity monitoring have allowed to quantify and assess biodiversity distribution and change across scales [5,6]. One of the most common approaches for assessing species distribution and dynamics has been the development of Species Distribution Models (SDMs) [7–9] (and references therein). Species Distribution Models (SDMs) can be defined as associative models for quantifying species-environment relationships, and are thus based on assessing the species’ ecological niche [10,11]. From a theoretical perspective [12], the species fundamental niche is a result of limiting abiotic factors at a broad geographical scale, typically related to climate or edaphic and geological properties [13]; whereas the realized niche is defined at a finer scale by habitat and biotic factors, mainly related to interspecific competition and dispersal ability, among others [14]. In the context of SDMs, the ecological niche is considered as a hypervolume in multivariate environmental space that depicts a species’ environmental requirements or limitations [12,15]. Once the ecological niche of a species has been defined through statistical functions, these can be applied to scenarios of climate or landscape conditions to project the future variation of the species’ distribution. However, the application of SDMs in conservation and management is still hampered by significant spatial and temporal biases (e.g. taxonomy errors, sampling overlapping, interpolations with insufficient data, inaccuracies in geo-referencing, etc.), both in species occurrence data, and in the set of predictive variables that represent the environmental variability [16].

One of the major drawbacks of species distribution modelling is that species occurrences are usually available at coarse resolutions [17], while their conservation and management within protected areas are needed at finer resolutions [18,19]. Another drawback is that many predictive variables are not measurable or available at the required resolution, so surrogates and interpolated data (e.g. from meteorological stations) have to be used instead [20,21]. Structural predictors derived from thematic cartography, such as land-cover variables, also hold limitations since they may not represent relevant landscape features nor the ecosystem processes relevant for the target species. Furthermore, both occurrence data and predictor variables can have inadequate or dissimilar spatial, thematic and/or temporal resolutions [22].

Earth observation techniques are becoming a fundamental toolkit to deal with some of these modelling biases and drawbacks [23–26]. Satellite remote sensing offers continuous and cost-effective measures of both abiotic and biotic factors across space and time, hardly quantifiable by other means [27,28]. Recent products derived from multispectral and hyperspectral sensors are playing a key role in the quantification, assessment and forecasting of biodiversity [29–31] by providing meaningful information to predict species distributions through climatic variables [32] as well as structural [33] or functional descriptors of ecosystems [34,35].

The use of satellite-derived ecosystem functional attributes (EFAs) as predictors in SDMs can have some advantages [35]. EFAs are descriptors of the overall ecosystem functioning [36,37], i.e. the exchanges of matter and energy between the biota and the physical environment, including, among others, indicators of productivity, seasonality and phenology of carbon gains [38–41]. At the regional scale over natural vegetation, EFAs are mainly driven by climate while they are more linked to land-cover and land-use at the local scale and with increasing human influence [39]. This way, EFAs offer an integrative and quicker view of ecosystem responses to environmental drivers and changes than structural or compositional attributes [42], linking species responses to pressures on ecosystem functioning and state [35], which is an advantage for implementing species monitoring programs [9,37,43]. Most importantly, EFAs can be monitored through remote sensing and derived globally under common protocols at relatively high temporal and spatial resolutions, which is particularly interesting for tracking and forecasting biodiversity changes when applied in SDMs [35]. In addition, EFAs can also help to overcome drawbacks of current climate and land-cover datasets, such as their difficulty to be updated [41] and the interpolation effects [32]. Alcaraz-Segura et al. [35] already provided a first illustration of the potential added-value of EFAs as meaningful species-level Essential Biodiversity Variables (EBVs) [30,44,45] to guide monitoring schemes for multiple protected species, due to their good predictive power in SDMs.

Several studies indicate that climate impacts on species distributions are most apparent at macro-scales [6,46], whereas land-cover may be a more important constraint than climate for species presence at the local scale [25,47]. The predictive role of EFAs in SDMs may thus be scale-dependent, as demonstrated for other abiotic and biotic predictors [48–50]. In addition, the relevance of EFAs for range shifts prediction is known to vary across groups of species [35]. Accounting for these potential caveats of the application of EFAs in SDMs is of high importance for real-world conservation challenges [51], particularly to assess the status and trends of species of conservation concern [52]. Building on this rationale and on the model-assisted biodiversity monitoring approach [9,35], the main goal of this study is to assess the predictive ability of remotely-sensed ecosystem functional attributes (EFAs) in Species Distribution Models (SDMs), and thereby to test their potential as Essential Biodiversity Variables (EBVs) for biodiversity monitoring and reporting. For this, we developed and applied a multi-scale modelling framework under two specific objectives: 1) to compare the performance and scale-dependence (in terms of spatial extent and resolution) of EFAs as predictors in SDMs, against traditional climate and land-cover predictors; and 2) to compare the spatial projections of habitat suitability derived from SDMs based on EFAs and on traditional predictors at various scales, and the corresponding implications for reporting the conservation status of protected species (e.g. under Art. 17 of the Habitats Directive [53]), and for guiding local conservation strategies. Our testable hypotheses and their underlying rationale are detailed below (‘Modelling framework’ section).

## Materials and methods

### Study areas

**Fig 1:**
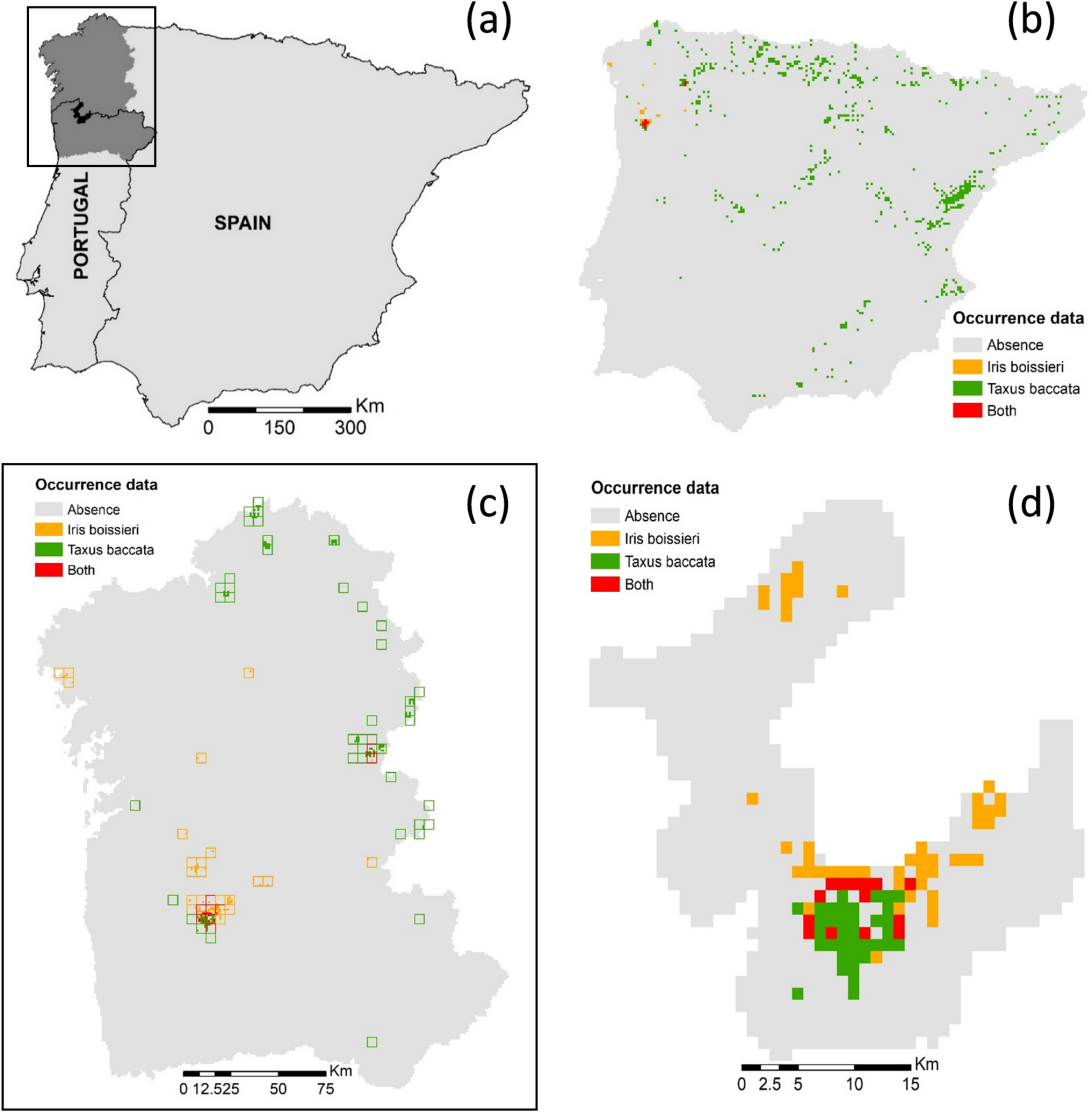
Study areas and species occurrence data. (a) The three nested study areas and occurrence data of target species (*Iris boissieri* and *Taxus baccata*) in (b) the Iberian Peninsula at 5km^2^ cell size, (c) the Iberian Northwest at 5km^2^ (empty squares) and 1km^2^ (filled squares), and (d) the Peneda-Gerês National Park at 1km^2^ cell size.

We tested our multi-scale approach using three nested study areas (Fig 1): The Iberian Peninsula (IP), the Iberian Northwest (Galicia in Spain, and northern Portugal; NW), and the Peneda-Gerês National Park, in Portugal (NP). We established the IP (581000 km^2^) as the biogeographic context of reference to fit sub-continental models, since its combination of natural history, geologic and topographic heterogeneity, and strong climatic gradients offers a wide range of environmental conditions for hosting a broad variety of endemic and rare plants [54]. Within the IP, we considered the northwest corner (NW), and within it the Peneda-Gerês National Park (NP), to fit regional and local models, respectively. The NW (48000 km^2^) is a diverse phytogeographic area with a diversified flora (ca. 2300 native species) dominated by Eurosiberian and Mediterranean elements, and with a large number of narrow endemics and biogeographic disjunctions [55]. Peneda-Gerês (700 km^2^) is a mountain range hosting more than 800 plant species, including various narrowly distributed endemics and other regionally rare species.

The Iberian Peninsula (Fig 1A and 1B) is characterized by a strong climatic gradient, from the rainiest and coldest areas with temperate climate in the north and northwest (Euro-Siberian region), to the driest and warmest areas in the south and southeast (Mediterranean region). This environmental context grants a high diversity of habitats and biotic communities. The Euro-Siberian (Atlantic) region holds vegetation types such as alpine natural and seminatural grasslands and heathlands, and forest ecosystems with alpine needleleaf coniferous and temperate broadleaf deciduous and semideciduous species; whereas the Mediterranean region is primarily represented by evergreen broadleaf and conifer canopy species in forest ecosystems, and a huge representation of shrub and herbaceous species able to resist the adverse conditions of long summer-drought periods. Landscapes in both regions have been largely transformed by human management, first through a combination of farming, grazing, fires, and firewood collection, more recently into specialized agricultural, agro-forestry or forestry production landscapes. Protected areas across the IP now hold most of its remaining mosaics of natural and seminatural vegetation.

The NW of the Iberian Peninsula (Fig 1C) is a mountainous territory with altitudes ranging from sea level to 2650 m, with slopes often over 25%. The climate is quite varied, but follows a general oceanic pattern with annual rainfall from 600 to 2000 mm (3000 mm in mountain areas) [56]. The average annual temperature ranges from 5°C in the highest mountains to 15°C in the lowland southern territories. In spite of the great influence of human activities, and the expansion of alien species for timber production and ornamental use, the current vegetation still holds similarities with the European Atlantic flora [57,58]. Deciduous forest dominated mainly by *Quercus robur* and *Fagus sylvatica* usually appear restricted to the top-half of the region, namely inside protected areas. Mixed forests of *Ulmus* sp., *Acer* sp., and *Salix* sp., among others, are mostly frequent at the southwest. Replacing forests, seral scrub of thorny bushes (*Cytisus* sp., *Ulex* sp. and other) are common across the region, as are meadows and other grasslands which play a relevant role in traditional agricultural systems. Towards south, the transition into the Mediterranean region is revealed by the occurrence (and dominance) of evergreen vegetation such as forests of *Quercus rotundifolia*, *Q*. *suber*, *Q*. *faginea* and scrub dominated by *Arbutus unedo* and other evergreen shrubs.

The Peneda-Gerês National Park (NP; Fig 1D) is a mountainous protected area located at the core of NW Iberia. The average annual temperatures range from 5°C in the highlands to 20°C in the valleys; whereas the total mean rainfall reaches 2000 mm per year (with more than 130 rainy days per year), and snowfall is frequent in the mountain tops. The steep topographic and climatic variations have produced a mosaic of vegetation types characteristic of Mediterranean, Euro-Siberian and Alpine environments. Deciduous oak forests of *Q*. *robur* and *Q*. *pyrenaica* are common throughout the Park in areas above 700m. Still, long-term grazing and the use of fire have facilitated the replacement of forests by pastures, heath and scrub (74% of the Park’s area). Forest plantations mainly of *Pinus pinaster* also occupy substantial areas. The highest rocky outcrops and remote areas are the main habitat for several endemic species, many of which are considered endangered and are under national and European protection programs.

### Test species and occurrence data

As test species, we focused on two vascular plants covered by the Habitats Directive (hereafter HD) and for which EU member-states hold regular reporting obligations (under Article 17 of the HD): the ‘Gerês lily’ (*Iris [Xiphion] boissieri* Henriq.; Annex IV), an endemic, narrow-ranged bulbous plant holding a ‘critically endangered’ conservation status (http://www.iucnredlist.org/details/162312/0), and the ‘European yew’ (*Taxus baccata* L.), indicator and dominant species of HD Annex I priority habitat type 9580* (‘Mediterranean *Taxus baccata* woods’). These two species sharply differ in terms of their distribution range (narrowly *vs*. widely distributed) and life-form (bulbous geophyte *vs*. tree), representing contrasting rarity types and thus different challenges for predictive niche modelling.

The Gerês-lily (*Xiphion boissieri* (Henriq.) Rodion = *Iris boissieri* Henriq.) is a narrow endemic bulbous plant of the *Iridaceae* family. It is listed in Annex IV of the HD and is restricted to mountainous areas of NW Iberian Peninsula. Portugal concentrates the largest populations of the species, especially in the Peneda-Gerês National Park, whereas in Spain it occurs as small populations in the neighbouring Sierras of Baixa Limia-Xurés and Santa Eufémia and in a few other mountains (https://eunis.eea.europa.eu/species/186604). It mainly colonizes small depressions with accumulation of coarse deposits, but it also occurs in low scrub and in crevices of granite outcrops, at elevations between 500 and 1500 meters [55]. The abandonment of pastoral systems is triggering vegetation succession and potentially reducing its area of suitable habitat [59].

The European yew (*Taxus baccata*) is a long-living tree native in most of Europe, with the southern limit of its distribution range in mountainous areas of the Mediterranean basin [60,61]. There is evidence of strong regression in southwest Europe, where *T*. *baccata* now occurs as small, isolated populations, making it a vulnerable species [62]. *T*. *baccata* woodlands may originate as a senescent or disturbed phase of deciduous woodlands, in which the species occurred as an understory tree. Yew stands can also be found along mountain streams where the trees can shelter from fire disturbance and from expansion of tall canopy broadleaved trees. Even if this habitat type is protected under the HD (https://eunis.eea.europa.eu/habitats/10239), there is a lack of knowledge about the distribution and conservation status of *T*. *baccata* woodlands in this southern edge of their range [63,64].

We used occurrence records for *I*. *boissieri* and *T*. *baccata* (presence-only dataset) to compute the response variables for SDM calibration. All georeferenced records were obtained from the Global Biodiversity Information Facility (http://www.gbif.org; accessed September 2016) with geographic accuracy equal to, or better than, 1 km^2^ spatial resolution. An additional set of 72 records resulting from a local (Peneda-Gerês) survey of *I*. *boissieri* performed in 2007 was added to this dataset. Subsequently, records were checked using GIS to detect georeferencing and species nomenclature errors. The final occurrence dataset was assumed to represent the whole (or most of the) geographic and environmental range of both species (cf. Fig 1). The final dataset for *I*. *boissieri* included records that ranged from 1992 to 2007, while for *T*. *baccata* they ranged from 1971 to 2016 (Table 1) (S1 and S2 Datasets).

**Table 1:** Occurrence data (number of grid cells) available per species at each combination of spatial resolution and spatial extent.

Spatial resolution	Spatial extent	Species distribution records	
		*Iris boissieri*	*Taxus baccata*
5km^2^	IP	30	440
5km^2^	NW	30	37
1km^2^	NW	91	139
1km^2^	NP	62	50

IP = Iberian Peninsula; NW = North-western Iberian Peninsula; NP = Peneda-Gerês National Park

To test our modelling approach at the three focal scales, the available records were then aggregated in two spatial grids with distinct cell size– 5 km^2^ for the sub-continental (IP) and the regional (NW) scales, and 1 km^2^ for the regional (NW) and the local (NP) scales. The test was thus conducted considering two dimensions of spatial scale: the resolution of the species records (1x1 km *vs*. 5x5 km grid cell size) and the spatial extent of the test area (IP *vs*. NW *vs*. NP).

### Modelling framework

**Fig 2:**
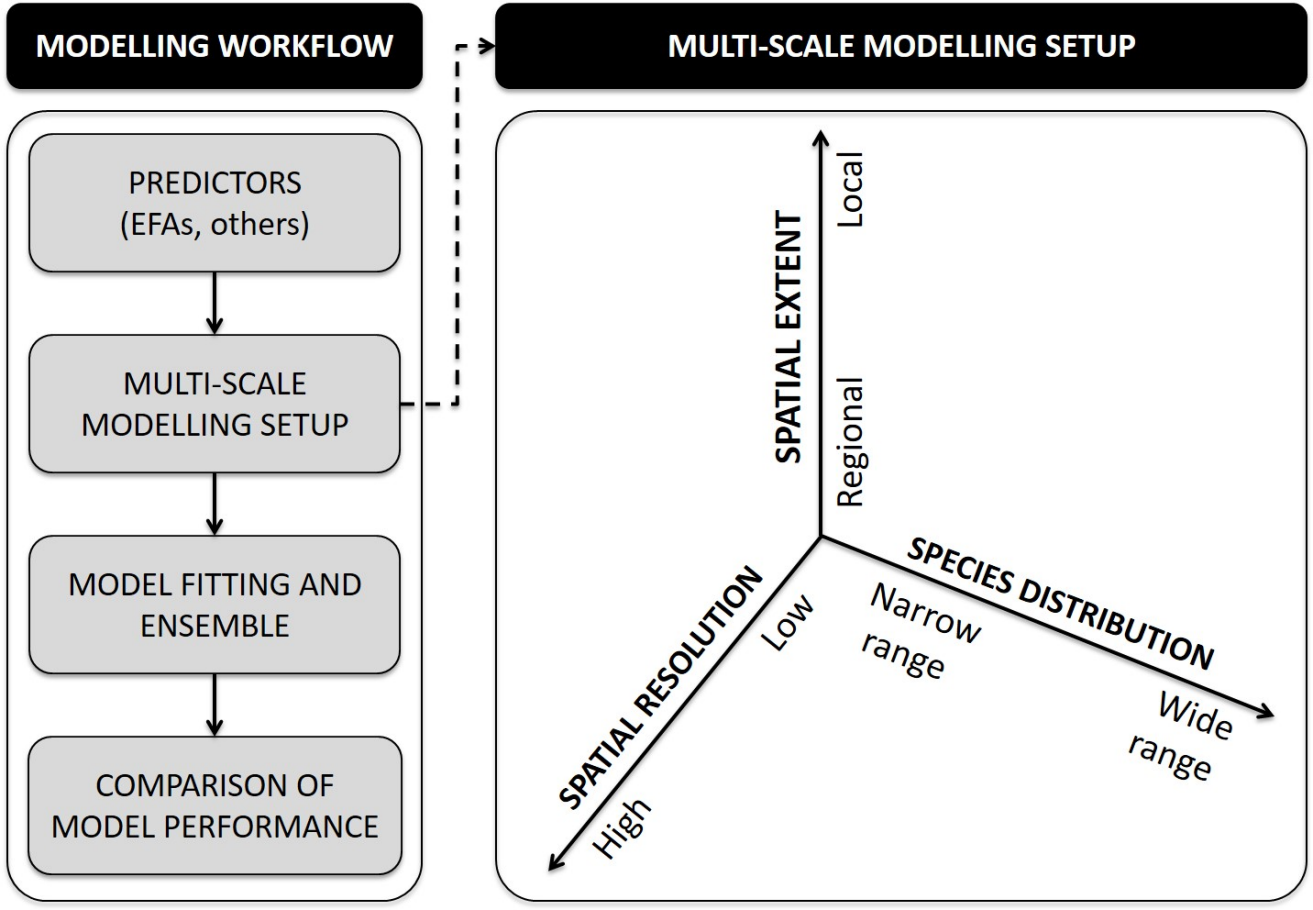
Multi-scale modelling framework. General framework to test the scale-dependence of the performance of satellite-derived Ecosystem Functional Attributes (EFAs) as predictors in Species Distribution Models (SDMs).

There is accumulated evidence of the importance of climatic and land-cover predictors in SDMs, their performance and scale-dependence [20,65]. Remote sensing can also provide a broad diversity of environmental descriptors [26] to SDMs, but there is still little knowledge of the predictive ability of the different variables, and of the effect of spatial extent and data resolution (but see [66,67]). To assess the predictive ability of remotely-sensed EFAs in SDMs, we established testable guiding hypotheses (Table 2) based on literature review and expert knowledge. To test these hypotheses, we developed a modelling setup (Fig 2) that involved two species, two spatial resolutions, three spatial extents (Fig 2), and four groups of predictors (Table 3): 1) interpolated climate data, 2) landscape composition, structure and diversity metrics, 3) remotely sensed proxies of vegetation functioning (EFAs), and 4) a combination of climate and landscape (land-cover) metrics. This modelling setup was also designed to facilitate the interpretation of EFA predictors in terms of climatic and land-cover constraints, and the identification of the most relevant predictors at each of the focal scales.

**Table 2:** Specific testable hypotheses for comparison of the performance and scale-dependence (in terms of spatial extent and resolution) of ecosystem functional attributes (EFAs) against traditional climate and land-cover datasets in Species Distribution Models (SDMs).

Hypotheses	Rationale		
H_1_	Given that EFAs capture the overall integrative response of the system to all environmental factors [35,38], remotely-sensed EFAs should perform as predictors in SDMs similarly or better than the combination of interpolated climatology grids plus land-cover data.		
H_2_	Being climate the main species driver at the regional scale and land-cover relatively more important at the local scale (e.g. [6]), the added-value of EFAs will also be scale-dependent.		
H_3_	As observed in previous studies [35], such scale-dependence should differ according to the species distribution range.	H_3.1_	For a narrowly distributed species, climate should perform better than EFAs at macro-scales and coarse resolutions, while they should similarly perform at local scales and fine resolutions.
		H_3.2_	For a widely distributed species, climate and EFAs should perform similarly both at macro-scales and coarse resolutions, and at local scales and fine resolutions.

**Table 3:** Final sets of predictors used to calibrate models. The description and attributes of the original datasets are also provided.

Set	Predictors		Code	Description	Units	Spatial resolution	Source
CLI	Mean Temperature of Wettest Quarter		TmWQ	The average temperature of the three consecutive months with the highest cumulative precipitation total.	°C*10	0.0083° (~1 km)	http://www.worldclim.org
	Mean Temperature of Driest Quarter		TmDQ	The average temperature of the three consecutive months with the lowest cumulative precipitation total.			
	Temperature Annual Range		TAR	The mean difference between the month’s maximum and minimum temperature over the twelve months of the year.			
	Precipitation of Wettest Month		PpWM	The total precipitation that prevails during the wettest month (with the highest cumulative precipitation total).	mm		
	Precipitation of Driest Month		PpDM	The total precipitation that prevails during the driest month (with the lowest cumulative precipitation total).			
	Precipitation Seasonality (Coefficient of Variation)		PS	The ratio of the standard deviation of the monthly total precipitation to the mean monthly total precipitation over the course of the year.			
LC	Composition	Agriculture	agric	Areas characterized by herbaceous vegetation that has been planted or is intensively managed for the production of food, feed, or fiber.	% area	0.00083° (~100 m)	http://land.copernicus.eu/
		Forest	forest	Areas characterized by tree cover, natural or semi-natural woody vegetation, generally greater than 6 meters tall.			
		Scrubs	scrubs	Areas characterized by natural or semi-natural woody vegetation with aerial stems, generally less than 6 meters tall, with individuals or clumps not touching to interlocking.			
		Bare soil	bs	Areas characterized by bare rock, gravel, sand, silt, clay, or other earthen material, with little (widely spaced and scrubby) or no "green" vegetation present regardless of its inherent ability to support life.			
	Diversity	Shannon's Diversity Index	SHDI	Proportion of the landscape occupied by a given patch class.			
	Structure	Mean Patch Area	AREAmean	The average mean surface of patches.			
EFAs	ALB Annual Maximum		ALBmx	The average between Daytime Black-Sky (Direct radiation) Shortwave Albedo and White-Sky (Diffuse radiation) Shortwave Albedo.	-	0.002° (~250 m)	https://lpdaac.usgs.gov/dataset_discovery/modis/modis_products_table/mod13q1
	EVI Annual Maximum		EVImx	The interannual mean of the EVI maximum.			
	EVI Annual Minimum		EVImn	The interannual mean of the EVI minimum.			
	EVI sine of the momentum of maximum		EVIdmxs	The momentum of the maximum green-up days of year decomposing it into the sine orthogonal vector related to springiness and autumness axis.			
	LST Standard Deviation		LSTsd	The interannual standard deviation of the Land Surface Temperature mean.	°C		
	LST Annual Minimum		LSTmn	The interannual mean of the Land Surface Temperature minimum.			

#### Environmental predictors

The set of predictors used for SDM calibration included (Table 3):

1. Bioclimatic variables (CLI) mainly related to temperature and precipitation regimes, from the WorldClim version 2 (1970–2000 period) database with a spatial resolution of 30 arc-seconds (~1 km) (http://www.worldclim.org; [68]). CLI predictors were selected for their predictive ability as well-known drivers of species distributions at broader scales [69]. The complete CLI dataset included 19 candidate predictors.
2. Several studies have shown the importance of landscape composition and configuration for predicting the distribution of species [70]. We therefore computed several landscape variables from the CORINE Land Cover 2006 (Label 3) database with a spatial resolution of 100 meters (http://land.copernicus.eu/pan-european/corine-land-cover). Composition metrics were computed as % of grid cell area covered by each LC class. We also computed spatial configuration metrics using the FRAGSTATS software (version 4.2) [71]. The complete landscape dataset (LC) included 61 composition and configuration candidate predictors.
3. Ecosystem Functional Attributes (EFAs). Three MODIS (Moderate Resolution Imaging Spectroradiometer) satellite-products were selected to describe three dimensions of ecosystem functioning: the Enhanced Vegetation Index (EVI) (MOD13Q1.006) as a surrogate for the carbon cycle dynamics, the Land Surface Temperature (LST) (MOD11A2.005) as a surrogate of sensible heat dynamics, and Albedo (MCD43B3.005) as a surrogate for the radiative balance [37], all for the 2001–2016 period at the original spatial resolution of 230m. We selected EVI instead of any other vegetation index (such as SAVI, ARVI, or NDVI) as an indicator of carbon gains since it is known to be more reliable in both low and high vegetation cover situations, and resistant to both soil influences and canopy background signals, and atmospheric effects on vegetation index values [72,73]. For these three dimensions of ecosystem functioning (EVI, LST and Albedo), we used Google Earth Engine [74] to derive the inter-annual mean of the following eight summary metrics of their seasonal dynamics: annual mean (surrogate of annual total amount), annual maximum and minimum (indicators of the annual extremes), seasonal standard deviation (descriptor of seasonality), and sine and cosine of the dates of maximum and minimum (indicators of phenology) [35,39,75]. EVI values ranged from -1 to 1, with healthy vegetation generally holding values between 0.20 and 0.80. Temperatures (LST) ranged from -25°C to 45°C, and Albedo values ranged from 0 to 1 (fresh snow and bare soil usually fall around 0.9). Sine and cosine of the maximum and minimum green-up days of the year are related to springiness/autumness and winterness/summerness, respectively [39,76]. Thus, values near +1 on the sine are in March/April, near –1 are in September/October, while values near +1 for the cosine are in December/January, –1 are in June/July. The complete EFAs dataset included 24 ecosystem functional attributes (8 metrics x 3 dimensions) as candidate predictors.

All datasets were re-projected to coordinate system WGS84/UTM zone 30 (http://spatialreference.org/ref/epsg/wgs-84) and resampled from their original spatial resolutions to the resolution of species occurrence records. Resampling was first done to a 1km^2^ for usage in the regional (NW) and local (NP) scale models, and to a 5 km^2^ cell size for the regional (NW) and sub-continental (IP) scale models. As highly correlated variables may hamper the fitting and validation of models [77], we conducted a multicollinearity analysis of datasets using the Spearman’s correlation coefficient and the Variance Inflation Factor (VIF) [78,79]. Considering further that the number of explanatory variables in the models influence both accuracy and predictive power [80], and that the species’ prevalence has a strong impact on model performance [81], we established that no more than *m/5* predictors should be included in each competing model for both the narrow-ranged and the wide-ranged species, where *m* is the number of occurrence records [82]. Therefore, since the minimum number of records was 30 (cf. Table 1), only six independent predictors with Spearman’s pairwise correlation <0.8 [83] and VIF < 4 (S1–S4 Figs and S5 and S6 Figs, respectively), and with the highest relative contribution per model extent and spatial scale combination in preliminary tests, were considered in model calibration (i.e. those listed in Table 3). The final set of predictors was defined considering both the results of the statistical tests and, when two or more predictors were highly correlated, only the one representing a more direct determinant of the ecology and distribution of the species was kept, based on expert judgement and scientific literature.

#### Modelling setup

We established three groups of models representing the effects of the three sets of predictors described above (climate, land-cover, and EFAs) on the distribution of the two test species (Table 4). Additionally, a combination of predictors related to climate and landscape composition/configuration was used in a fourth set of models, using the same predictors used for the CLI and LC models.

**Table 4:** Rationale for the four groups of models included in the modelling setup.

Datasets	Rationale	References
CLI	Climatic gradients (CLI) usually govern species distributions at global to regional scales. However, climate may not affect equally the distribution of narrow-ranged and wide-ranged species.	[20] [84] [69]
LC	Land-cover (LC) mainly affects species occupancy patterns at the landscape and local scales. Landscape composition and structure have been used for predicting species diversity as well as the distribution and abundance of individual species.	[85] [25] [86]
CLI + LC	Climate (CLI) and land-cover (LC) are known to influence species distributions at various scales, therefore models combining climate and land-cover predictors are due to provide robust predictions of those distributions. Climate and land-cover are also drivers of EFAs, and so CLI+LC models are assumed to approach, from a structural perspective, the potential effects of EFAs on those distributions.	[87] [88] [89]
EFAs	The annual metrics derived from EVI time-series are closely related to the dynamics of ecosystem carbon gains, and therefore, to net primary productivity. These functional attributes (EFAs) allow capturing most of the variability in phenology, seasonality and productivity, holding high predictive power over species distributions at the local and regional scales.	[38] [90] [40]

To analyse and rank model performance, we conducted a modelling workflow testing the effect of the three focal drivers of model performance (predictor set, spatial extent, and spatial resolution), and for the narrow-ranged and the wide-range species. Thus, we compared model performance in three sets of tests: 1) different predictor sets (CLI, LC, CLI+LC, EFAs), at same pixel size and spatial extent; 2) different pixel sizes (1 km^2^ *vs*. 5 km^2^), for the same spatial extent and with the same predictor set; and 3) different spatial extents (IP *vs*. NW *vs*. NP), at the same pixel size and for the same predictor set.

#### Model fitting and evaluation

We fitted Species Distribution Models (SDMs) and obtained spatial projections under an ensemble-forecasting framework implemented on *biomod2* package ([91]; available at http://cran.r-project.org/web/packages/biomod2/index.html). The ensemble-forecasting framework has been established as a powerful tool for analysing species-environmental relationships [92]. Models were fitted using all 10 modelling techniques available in *biomod2*, for each set of models and using default parameters. Since algorithms require the input of (pseudo)absences [93], and since true-absence data were not available for the target species, pseudo-absences were generated by randomly assigning unoccupied grid cells each the study region, with the following constraints: 1) generating the same number of pseudo-absences as of presences to avoid potential bias caused by different levels of prevalence in the presence/absence datasets [94]; and 2) defining a minimum distance between pseudo-absences, corresponding with each grain size (5 km and 1 km), and without overlapping with presences [95], to avoid spatial autocorrelation and in order to cover the different ecological conditions in each study area. Uncertainty was controlled by generating 30 different sets of pseudo-absences for each species and running the whole process 30 times, resulting in 9300 individual models produced for each combination of species, spatial extent and grain size.

Model accuracy was were measured as the Area Under the Curve (AUC) of receiver operator characteristic (ROC) curves. AUC is a robust threshold-independent measure of a model’s ability to discriminate presence from absence [79,96], ranging between 0 and 1 (measures below 0.7 were considered poor, 0.7–0.9 moderate, and > 0.9 good). The resulting models with the highest AUC median, among those satisfying the condition ≥ 0.7 [97], were selected. We used AUC median values for excluding models with lower scores, for the binary transformations of model probability predictions, and for building the ensemble predictions. Differences between the mean predictive ability of the best models in terms of AUC were also compared using the Tukey's HSD (honest significant difference). Additionally, we used the True Skill Statistic (TSS) median values of the ensemble models as a threshold-dependent measure of model accuracy [98]. Since TSS ranges from 0 or less (agreement no better than random classification) to 1 (perfect agreement between predictions and observations), we considered TSS values < 0.4 poor, 0.4–0.8 useful, and > 0.8 good to excellent. Model evaluation was based on cross-validation, with the species datasets divided into 80% of the records for model calibration and 20% for model evaluation. To transform predicted probabilities into suitable/unsuitable areas, we used a threshold minimizing the straight-line distance between the receiver operating curve plot and the upper-left corner of the unit square [99].

### Comparison of best predictors and spatial projections across sets of models

The importance of each predictor in the final ensemble models, ranging between 0 (no importance) and 1 (high importance), was computed as described in [92]. We compared the relative importance of predictors across the four sets of models (CLI, LC, CLI+LC, and EFAs). Only predictors with importance > 0.1 were considered. To facilitate ecological interpretability of the effect of the most relevant predictors, we analysed the response curve (maximum sensitivity range and sense of the slope) of the best predictor (the one with the highest importance) in EFA-based models and in the best models based on CLI and/or LC predictors.

To assess the usefulness of EFA-based SDMs in reporting the conservation status of protected species (e.g. Article 17 HD), we compared the spatial projections of habitat suitability derived from EFA-based models and from CLI/LC-based models. For pairwise comparisons between projections obtained at each combination of spatial extent (IP, NW and NP) and spatial resolution (1km and 5km) for each species, were used the improved fuzzy Kappa algorithm implemented in the Map Comparison Kit version 3.2.3 [100] for categorical maps. This algorithm expresses the mean agreement between two maps, compared to the expected agreement from random relocation of all grid squares in both maps, ranging between 0 (totally different maps) and 1 (identical maps). It considers the fuzziness of the location, for near ‘grid square-by-grid square’ agreement, while also accounting for autocorrelation in the changes amongst maps [100,101]. We also tested the agreement between continuous prediction maps using the Spearman’s correlation coefficient and the Moran’s I index for spatial autocorrelation.

All analyses were performed in the R software version 3.4.1 [102] available at CRAN (http://cran.r-project.org/). QGIS version 2.18.11 [103] and ArcGIS version 10.2 [104] were used for managing and representing spatial data and projections.

## Results

### Comparison of model performance across species and scales

**Fig 3:**
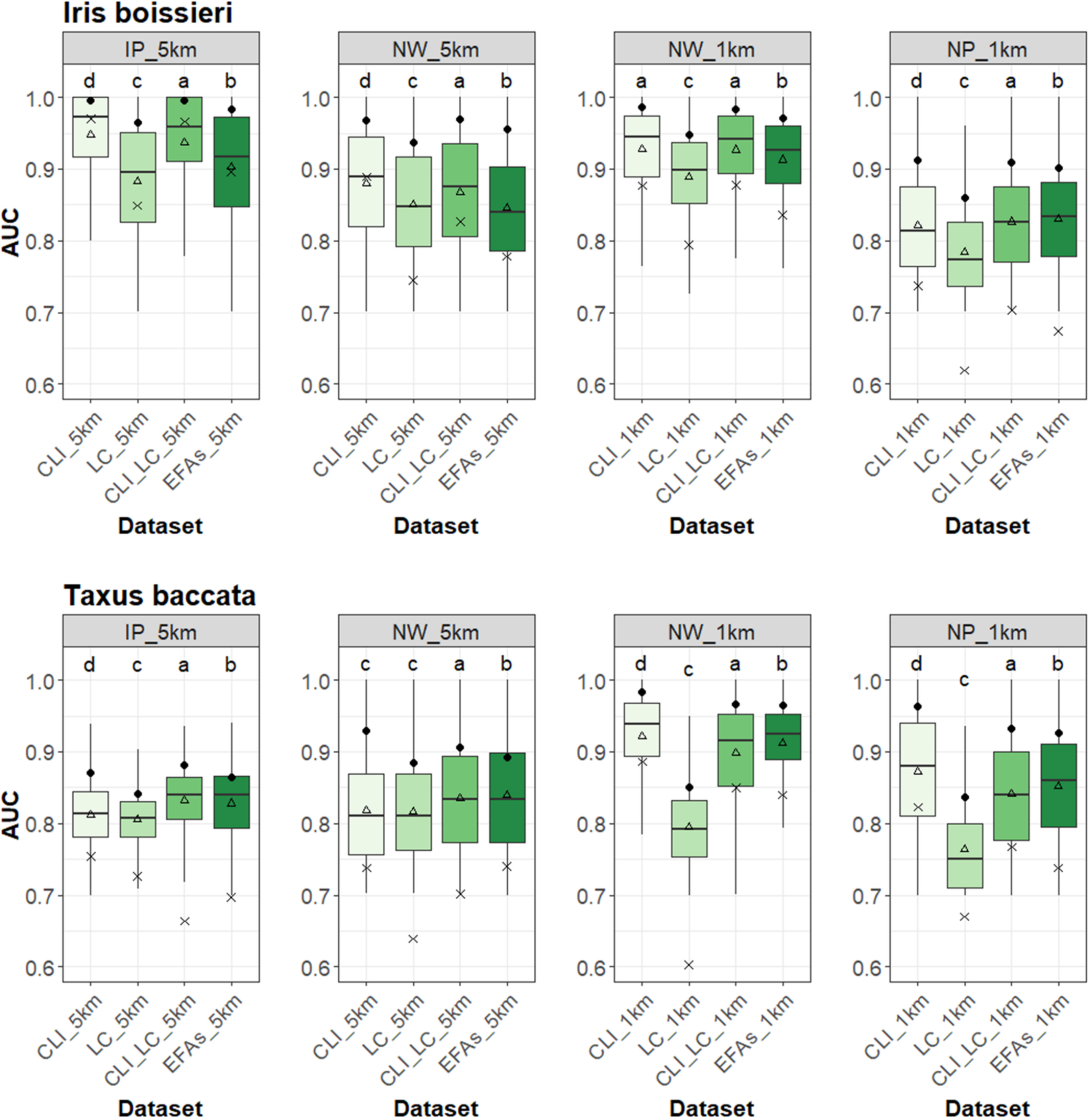
Comparison of relative performance of the Area Under the Curve (AUC) between traditional (climate and land-cover)-based and satellite-derived Ecosystem Functional Attribute (EFA)-based models across all scale combinations and test species. Performance of individual models (boxplots) showing the AUC_median_, two hinges (first and third quartiles), and two whiskers of each model filtered at AUC≥0.7 (empty-triangle signs represent the AUC_mean_). Filled-circle dots and crosses represent the AUC_median_ and the TSS_median_, respectively, of the ensemble models. Different letters indicate significant differences among models (multiple comparisons of means were performed using Tukey's test at the 0.05 significance level).

Overall, models exhibited very good performance as measured by AUC_median_ (Fig 3). CLI-based models, individually or in combination with LC, showed the highest values in all cases (from 0.882±0.059 to 0.995±0.083), though EFA-based models held only slightly lower performance (from 0.881±0.072 to 0.983±0.125) (Table 5). TSS_median_ values were above 0.6 in all cases, ranging from 0.66±0.03 to 0.97±0.17 (S7 and S8 Figs).

**Table 5:** Summary table of performance of the best ensemble models based on traditional (climate -CLI- and/or land-cover -LC-) and on satellite-derived Ecosystem Functional Attribute (EFAs), considering those individual models filtered at Area Under the Curve (AUC) ≥0.7. The AUC_median_±IQR (Inter Quartile Range) and the top variables above a threshold of importance contribution (% >0.10) are showed per species, extent, and spatial resolution. See complete names of variables and extents in Table 3.

Extent	Spatial resolution	*Iris boissieri*					*Taxus baccata*				
		Model	AUC_median_ (±IQR)	TSS_median_	Top variables	% variable contribution	Model	AUC_median_ (±IQR)	TSS_median_	Top variables	% variable contribution
IP	5km	CLI	0.995±0.083	0.97±0.16	PpWM	0.67	CLI+LC	0.882±0.059	0.66±0.09	agric	0.52
					PpDM	0.13				TmDQ	0.28
		EFAs	0.983±0.125	0.9±0.25	EVIdmxs	0.51	EFAs	0.881±0.072	0.7±0.1	LSTmn	0.47
					LSTsd	0.31				LSTsd	0.26
					ALBmx	0.17				EVIdmxs	0.11
NW	5km	CLI	0.968±0.125	0.89±0.25	TmDQ	0.71	CLI	0.929±0.113	0.74±0.17	TmDQ	0.67
					PS	0.13				PpDM	0.12
					PpWM	0.11				PS	0.11
		EFAs	0.955±0.118	0.78±0.17	EVImn	0.55	EFAs	0.927±0.125	0.74±0.2	EVImn	0.64
					EVIdmxs	0.16				EVIdmxs	0.29
					EVImx	0.11					
	1km	CLI	0.986±0.084	0.88±0.11	TmDQ	0.79	CLI	0.983±0.074	0.89±0.15	TmDQ	0.53
										TAR	0.14
					PpWM	0.16				PS	0.12
										TmWQ	0.1
		EFAs	0.971±0.08	0.84±0.16	EVImn	0.54	EFAs	0.964±0.072	0.84±0.11	EVImn	0.62
					EVImx	0.17				LSTsd	0.28
					LSTmn	0.12					
NP	1km	CLI+LC	0.909±0.104	0.7±0.29	TmDQ	0.69	CLI	0.963±0.13	0.82±0.2	TmWQ	0.67
					agric	0.27				PpWM	0.19
										PS	0.11
		EFAs	0.902±0.104	0.68±0.25	EVImn	0.49	EFAs	0.931±0.115	0.74±0.2	EVImx	0.52
					LSTmn	0.21				EVImn	0.4

The ensemble AUC_median_ of EFA-based models for the narrow-ranged species (*Iris boissieri*) was always >0.9 in two of the four scale combinations (Table 5, Fig 3). IP-5km and NW-1km were the best scale combinations for this species (AUC_median_ of 0.983±0.125 and 0.971±0.08, respectively). In both cases, performance of EFA-based model performance was statictically comparable to that of CLI- and CLI+LC-based models. For the other two combinations, NW-5km and NP-1km, AUC_median_ values of EFA-based models were also above 0.9 (0.955±0.118 and 0.902±0.104, respectively) (Fig 3). LC-based models held the worst performances in all scale combinations for *Iris boissieri*.

The ensemble AUC_median_ of EFA-based models for *Taxus baccata* was lower than for *I*. *boissieri*, and ranged from 0.8 (IP-5km and NW-5km) to 0.9 (NW-1km and NP-1km) (Fig 3). The best scale combinations of EFA-based models for this wide-ranged species were for the NW and NP extents at 1km resolution (AUC_median_ of 0.964±0.072 and 0.931±0.115, respectively, statistically comparable to CLI- and CLI+LC-based models; Table 5). The other two scale combinations (IP-5km and NW-5km) also held AUC_median_ values for EFA-based models above 0.8 (Fig 3). LC-based models again held the worst performances in all scale combinations (Fig 3).

### Predictor importance and response curves

The mean contribution achieved by each predictor (only those ≥ 10%) for the ensemble models based on traditional (CLI and/or LC) predictors and on EFAs is summarized in Table 5 (see S9 and S10 Figs for predictor importance across all individual models and for each target species). Overall, climate predictors (temperature and precipitation) held the highest importance at the several scale combinations, while predictors related to productivity and phenology were the most important in EFA-based models.

**Fig 4:**
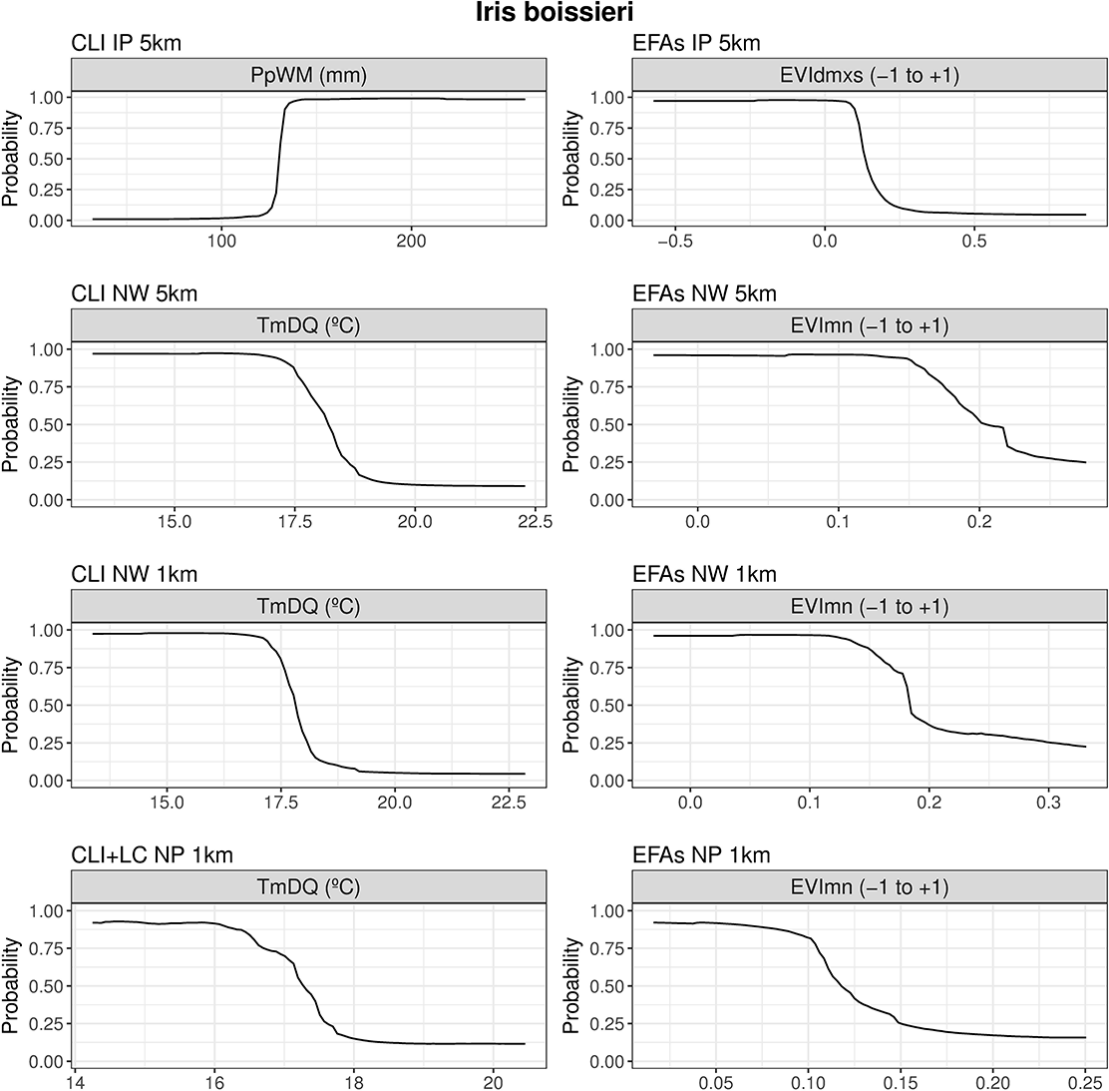
Response curves of predicted habitat suitability for *Iris boissieri* to the most important predictors. Response curves for predictors with the highest importance in traditional (climate and land-cover)-based (left) and Ecosystem Functional Attribute (EFA)-based (right) ensemble models for *Iris boissieri* at all combinations of spatial extents and resolutions.

For *Iris boissieri*, Precipitation of Wettest Month (PpWM) was the most important predictor in CLI/LC-based models at the largest and coarsest scale (IP-5km) (Table 5), whereas summer temperatures (TmDQ) held high predictive power at more local and finer scales (NW-5km, NW-1km and NP1-km). Land-cover (namely ‘agric’) was only important at the most local and finest scale combination (NP1-km). In EFA-based models, EVIdmxs (summerness of the growing season peak) was among the most important predictors at the largest and coarsest scales (IP-5km and NW-5km) (Table 5), while EVImn (minimum productivity) was an important predictor at more local and finer scales (NW-5km, NW-1km and NP1-km). Species response curves to the most important predictors (Fig 4) revealed that *I*. *boissieri* is distributed in areas of high precipitation and cool summer temperatures. From and ecosystem functioning viewpoint, the species occurs in areas where the growing season peak occurs in summer and with low productivity during the winter.

**Fig 5:**
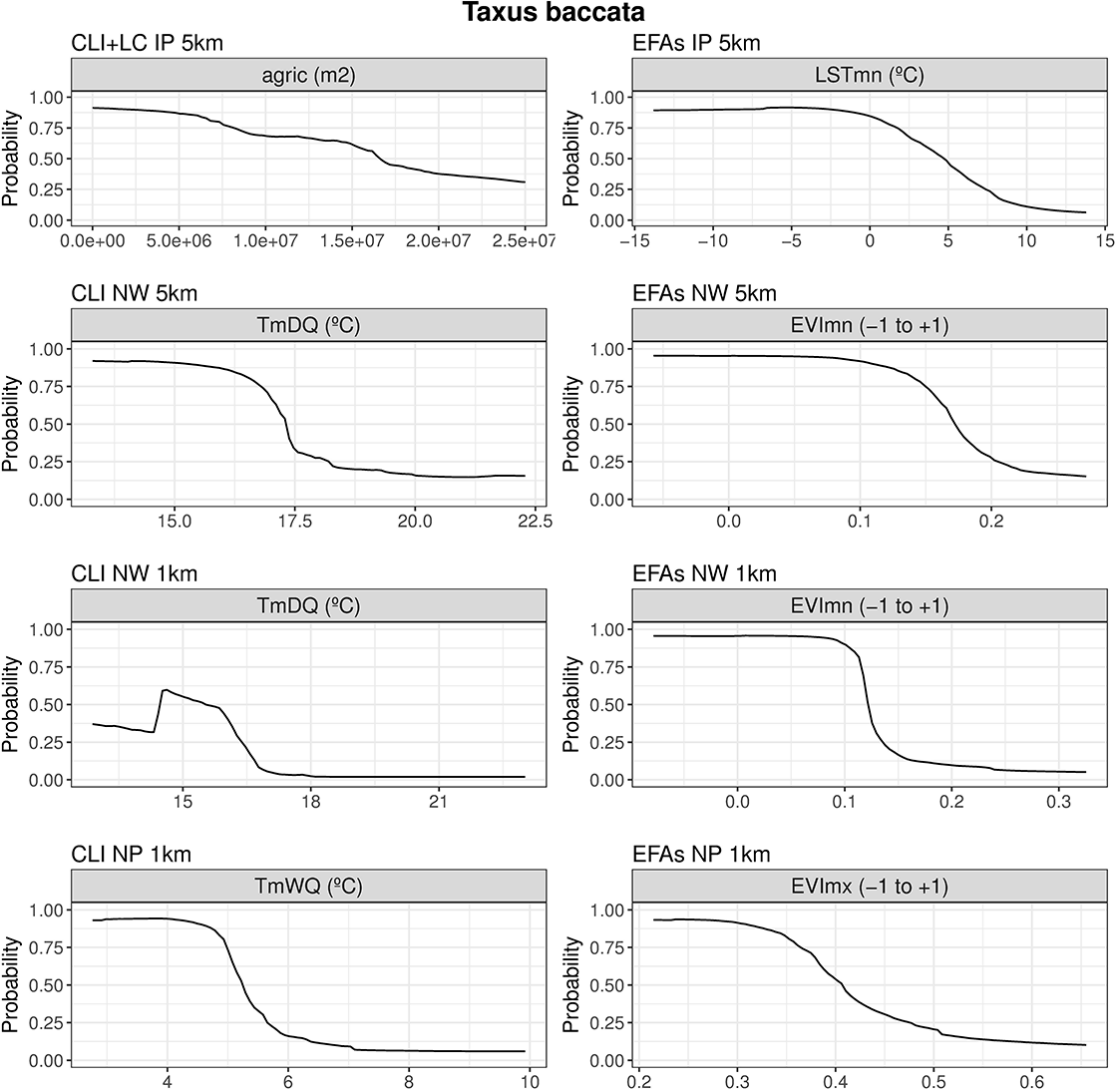
Response curves of predicted habitat suitability for *Taxus baccata* to the most important predictors. Response curves for predictors with the highest importance in traditional (climate and land-cover)-based (left) and Ecosystem Functional Attribute (EFA)-based (right) ensemble models for *Taxus baccata* at all combinations of spatial extents and resolutions.

For *Taxus baccata*, agricultural land-use (‘agric’) was the most important predictor in CLI/LC-based models at the largest and coarsest scale (IP-5km) (Table 5). However, climate was the main driver of the species distribution at more local and finer scales: summer temperatures (TmDQ) at regional and finer scales (NW-5km and NW-1km) and while winter temperatures (TmWQ) at the most local and finest scale combination (NP-1km). In EFA-based models, LSTmn, representing surface temperatures during the coldest month, was the most important predictor at the largest and coarsest scale (IP-5km) (Table 5), whereas EVI, our surrogate for productivity dynamics, was more informative at more local and finer scales. Thus, EVImn (minimum productivity) was an important predictor at several scale combinations, while EVImx (maximum productivity) was especially important at the most local and finest scale (NP-1km). Species response curves to the most important predictors (Fig 5) revealed that the occurrence of *T*. *baccata* is more likely in areas with cool temperatures and under lower agricultural management, such as in remote mountain areas. Response curves to the most important EFA predictors confirmed that the species is mainly distributed in areas of low surface temperatures and with low productivity during the winter.

### Spatial projections of habitat suitability

**Fig 6:**
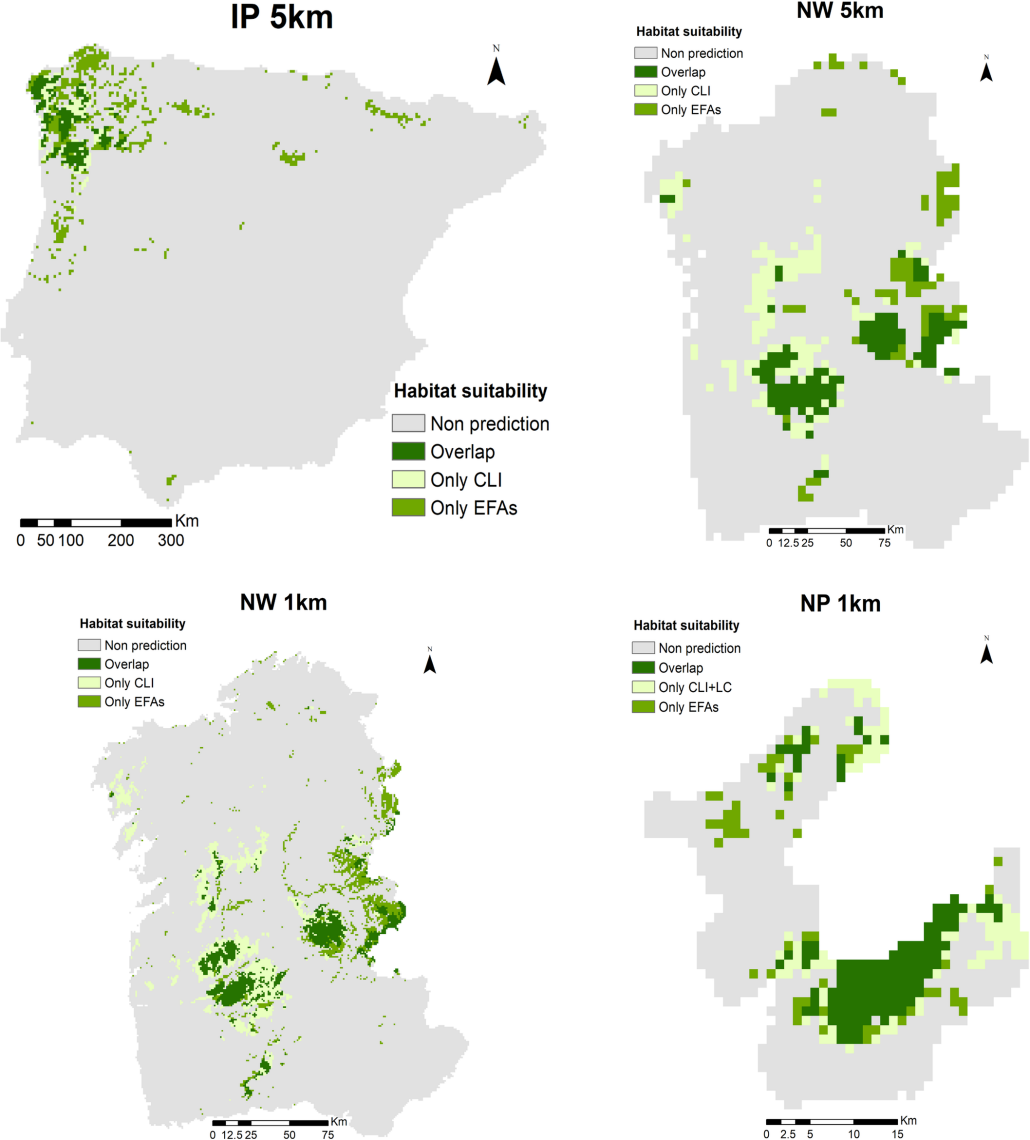
Spatial projections of habitat suitability for *Iris boissieri* derived from Species Distribution Models (SDMs) based on traditional predictors (climate and land-cover) and on satellite-derived ecosystem functional attributes (EFAs). Overlay maps of current potential presence-absence distributions predicted using an ensemble modelling approach per combination of spatial extent (IP, NW and NP) and resolution (1km and 5km) for *Iris boissieri*. IP: Iberian Peninsula; NW: Northwest IP; NP: Peneda-Gerês National Park.

**Fig 7:**
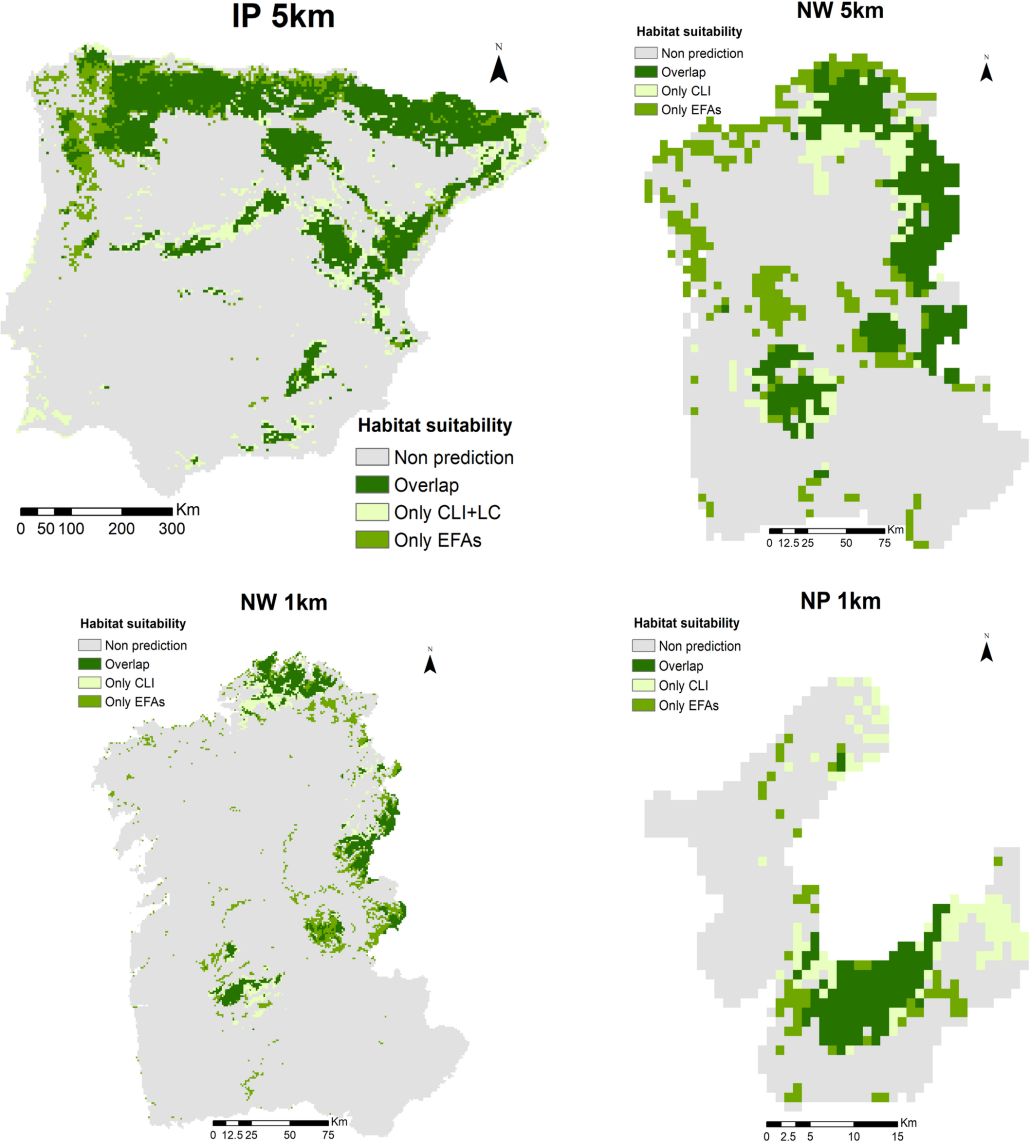
Spatial projections of habitat suitability for *Taxus baccata* derived from Species Distribution Models (SDMs) based on traditional predictors (climate and land-cover) and on satellite-derived ecosystem functional attributes (EFAs). Overlay maps of current potential presence-absence distributions predicted using an ensemble modelling approach per combination of spatial extent (IP, NW and NP) and resolution (1km and 5km) for *Taxus baccata*. IP: Iberian Peninsula; NW: Northwest IP; NP: Peneda-Gerês National Park.

Overall, similarity of spatial projections between CLI/LC-based and EFA-based SDMs for the narrow-ranged species (*Iris boissieri*) was highest at more local and finer scales (Table 6; Fig 6), while the greater similarities for the wide-ranged species (*Taxus baccata*) were found at larger and coarser scales (Table 7; Fig 7). Spatial autocorrelation (Moran’ I) of predicted habitat suitability was generally higher in projections from CLI/LC-based models than from EFA-based models (for the same species at scale combination) (Tables 6 and 7). In general, habitat suitability projections from EFA-based models were more conservative in terms of predicted suitable pixels (see S11 and S12 Figs).

**Table 6:** Comparison of spatial projections from traditional (CLI/LC-based) models and from Ecosystem Functional Attribute (EFA-based) models for *Iris boissieri* at all scale combinations. The proportion of predicted suitable area is shown in brackets. IP: Iberian Peninsula; NW: Northwest IP; NP: Peneda-Gerês National Park.

Extent	Spatial resolution	*Iris boissieri*						
		Model	Fuzzy Kappa	Spearman’s *ρ*	Moran’s I	Area (km^2^)		
						Partial	Overlaid	Total
IP	5km	CLI	0.561	0.43***	0.75	4425(14.84%)	7925(26.6%)	29800
		EFAs			0.56	17450(58.55%)		
NW	5km	CLI	0.628	0.51***	0.66	3225(40.31%)	2900(36.25%)	8000
		EFAs			0.55	1875(23.43%)		
	1km	CLI	0.543	0.41***	0.85	2483(42.62%)	1680(28.83%)	5826
		EFAs			0.63	1663(28.54%)		
NP	1km	CLI+LC	0.666	0.64***	0.63	89(32.24%)	134(48.55%)	276
		EFAs			0.57	53(19.2%)		

Note

*** means significance level at p < 0.001

**Table 7:** Comparison of spatial projections from traditional (CLI and/or LC-based) models and from Ecosystem Functional Attribute (EFA-based) models for *Taxus baccata* at all scale combinations. The proportion of predicted suitable area is shown in brackets. IP: Iberian Peninsula; NW: Northwest IP; NP: Peneda-Gerês National Park.

Extent	Spatial resolution	*Taxus baccata*						
		Model	Fuzzy Kappa	Spearman’s *ρ*	Moran’s I	Area (km^2^)		
						Partial	Overlaid	Total
IP	5km	CLI+LC	0.799	0.76***	0.74	33850(20.21%)	104725(62.54%)	167450
		EFAs			0.78	28875(17.24%)		
NW	5km	CLI	0.588	0.63***	0.74	2775(17.29%)	7550(47.04%)	16050
		EFAs			0.61	5725(35.67%)		
	1km	CLI	0.659	0.48***	0.76	930(20.12%)	1731(37.46%)	4620
		EFAs			0.61	1959(42.4%)		
NP	1km	CLI	0.539	0.66***	0.75	92(36.07%)	108(42.35%)	255
		EFAs			0.61	55(21.57%)		

Note

*** means significance level at p < 0.001

For *Iris boissieri*, the most significant correlation among continuous maps of habitat suitability was found between the combination of CLI+LC and LC-based models, and EFA-based models (r = 0.64; P < 0.001), at local and finer scale combination (NP1-km). This agreement was supported by the improved fuzzy Kappa values between these two maps (Fuzzy Kappa = 0.666) (Table 6). The spatial autocorrelation (Moran’ I) of the habitat suitability maps was positive for both CLI/LC-based and EFA-based models at all scale combinations. In terms of predicted area, EFA-based models predicted more partial suitable area than CLI-based models at the largest and coarsest scale combination (IP-5km), while CLI and the combination of CLI+LC gained more predicted power at more local, coarse and finer scale combinations (NW-5km, NW-1km and NP-1km) (Fig 6). The greatest overlaid area (48.55%) between the combination of CLI and LC was found at local and finer scale combination (NP-1km).

For *Taxus baccata*, the most significant correlation among continuous maps of habitat suitability was found between the combination of CLI+LC-based and EFA-based models (r = 0.76; P < 0.001), at larger and coarser scale combination (IP-5km). This agreement was supported by the fuzzy Kappa values between these two maps (Fuzzy Kappa = 0.799) (Table 7). The spatial autocorrelation (Moran’ I) of the habitat suitability maps was positive for both CLI/LC-based and EFA-based models at all scale combinations. In terms of predicted area, EFA-based models predicted slightly less partial suitable area than combined CLI+LC-based models at the largest and coarsest scale combination (IP-5km), while gained more predicted power at more local, coarser and finer scale combinations (NW-5km and NW-1km) (Fig 7). The partial predictions of the CLI-based models were higher than EFA-based predictions at local and finer scale combination (NP-1km). The greatest overlaid area (62.54%) between the combination of CLI+LC-based and EFA-based projections was found at regional and larger scale combination (IP-5km).

## Discussion

Traditionally, multi-scale approaches to examine scale-dependent effects on species response to environmental heterogeneity have focused on classical predictors such as climate and land-cover [46,67], but far less on ecosystem functional variables or remote sensing data [66]. Building on previous studies that explored the predictive value of satellite-derived ecosystem functional attributes (EFAs) [35], here we tested additional variables such as Albedo and Land Surface Temperature. We also tested whether EFA-based SDMs performance was scale dependent. We also tested whether the performance of EFA-based SDMs was scale-dependent, and compared it with climate and land-cover predictors at several spatial resolutions and extents, for a narrow-ranged species (*Iris boissieri*) and for a wide-ranged species (*Taxus baccata*).

Overall, our results showed that EFAs perform as good as the combination of climate plus land-cover as predictors in SDMs. For both groups of predictors, model performance and the most important predictors showed some variation across scales and species. The EFA-derived habitat suitability maps were consistent with those derived from climate plus land-cover, but with the advantage that EFAs provide ready-to-use and easily updatable information on suitable habitat conditions. Altogether, our results reinforce the use of satellite-derived EFAs related to the carbon cycle and the energy and radiation balance as meaningful Essential Biodiversity Variables (EBVs) of Ecosystem Function and for reporting species conservation status [35].

### Model performance and scale-dependence

For all scale combinations under analysis, EFA-based ensemble models showed almost the same performance (0.019% lower on average) than models based on climate or on a combination of climate and land-cover. Such small difference in performance (see Table 5, Fig 3) confirm that satellite-derived EFAs perform similarly to the combination of interpolated climatology grids plus land-cover data across scales and species ranges (hypothesis H_1_). Satellite descriptors of ecosystem functioning not only showed a similar performance to climate predictors in SDMs (as in [35,105]), but also to the combination of climate and land-cover, supporting the idea that EFAs capture an integrative response to multiple environmental drivers [25,39].

We found a relatively small scale-dependence of the predictive ability of satellite-derived EFAs (hypothesis H_2_), with model performance increasing towards higher spatial resolutions (NW-5km *vs*. NW-1km), larger extents of analysis (IP *vs*. NW *vs*. NP), and smaller species range (*Iris vs*. *Taxus*). Consistently with previous studies, our results also showed that the performance of SDMs based on traditional predictors increases at finer spatial resolutions [106] and with smaller number of records [81,107]. Such differences in performance agree with the importance of combining climate (a major species driver at the regional scale) and land-cover to achieve robust predictions at the local scale (e.g. [6]). In addition, performance of EFA-based SDMs was better for the narrowly distributed than for the widely distributed species at larger and coarser scales (as observed in [35] at the same spatial resolution and extent).

Satellite-derived variables are known to improve SDMs performance compared to other predictors [25], at various spatial extents and resolutions [108]. Our results show that the explanatory power of EFAs, compared to that of traditional predictors, differed according to the spatial extent, spatial resolution, and species distribution range (hypothesis H_3_). For our narrowly distributed species, the similarity in average performance between models based on EFAs, and the best models based on traditional predictors, increased from larger and coarser scales (0.012% difference) to local and finer scales (0.007% difference). However, for the widely distributed species, this similarity in average performance decreased from larger and coarser scales (0.001% difference) to local and finer scales (0.03% difference).

Overall, our results confirmed the known predictive ability of the satellite-derived EFAs at coarse resolutions [35], and expanded it to finer scales, even at the protected area level. This provides a local scale refined prediction of species potential distribution and habitat suitability that is closer to the scale required for management actions [6,109,110]. Such predictions could be further improved thanks to the availability of EFAs at even finer spatial resolutions (e.g., MODIS at ~250 m, LANDSAT at ~30m or Sentinel-2A at ~10m) and higher temporal resolutions (MODIS or the combination of LANDSAT plus Sentinel-2A), which is still a major constraint when using climate interpolated surfaces and land-cover data [35]. Furthermore, the use of EFAs in SDMs allows the interpretation of the determinants of species distributions from a functional perspective, considering features such as annual amount, seasonality and phenology of carbon gains, land surface temperature, and albedo. As new satellite products become available, EFAs could be expanded to other dimensions of ecosystem functioning such as evapotranspiration, soil moisture, disturbance, etc. [37,39].

Still, in spite of the limitations of the interpolated climate data and low-resolution land-cover data, models combining those predictors provided similar or slightly better performances than EFA-based models across scales and species ranges. Therefore, we assume that using high-resolution and updated spatial climate data and recent land-cover classifications derived from remote sensing, as well as ancillary edaphic and geological data, could further improve their performance [32,34]. For instance, the fusion of radar and optical remote sensing data generally leads to increased accuracy of the land-cover maps [111]. The synergy between high-resolution radar, optical (e.g. Sentinel-1 and Sentinel-2) and LIDAR data could also be used to inform on important aspects of habitat structure and function such as structural complexity of the canopy [112], or soil moisture mapping [113], which could help to improve model accuracy. Furthermore, the continuous updating of current climatic and land-cover datasets would complement the information provided by the integrative response of satellite-derived ecosystem functional variables (EFAs) [32,114]. This combination would improve our ability to forecast [35] the effects of environmental change on ecosystem function and structure, to monitor biodiversity hotspots, or to model habitat quality at regional and local scales [34,105].

### The role of EFAs in capturing both climate and land-cover effects in SDMs

In this study we also aimed to identify the most relevant variables in the models based on EFAs or on climate/land-cover predictors, comparing those variables across scales and species. As often found (e.g. [6,69,85,115]), our results showed that climate was the main driver of species distribution at all combinations of spatial extent, spatial resolution and target species, while land-cover achieved good performance only in combination with climate. Overall, temperature and precipitation provided the highest contributions for models based on traditional predictors across scales. We observed an increasing dominance of the temperature over precipitation variables from regional and coarser to local and finer scales in determining the species’ climatic range. Predictors related to productivity and phenology were the most important in EFA-based models (e.g. [24]), suggesting that remotely-sensed descriptors of the carbon cycle were the most capable of capturing relevant aspects of ecosystem functioning [35,50,116] for both narrowly and widely distributed species.

Our results revealed that, from regional and coarser scales to local and finer scales, our narrow-ranged species (*Iris boissieri*) tends to occur in areas characterized by cool temperatures and high precipitation during the summer season, which translates into a maximum primary productivity in summer and minimum in winter (see Table 5; Fig 4). These results match with our observations that the species is most often found at high elevations, in sparsely vegetated landscape mosaics dominated by scrub, and grasslands. In addition, the aerial part disappears as the summer advances, and only the underground part (the bulb) remains in a latent state during the less favorable season, taking advantage of local deposits of organic matter in the low, fire-prone scrubland typical of upper elevations [55]. The greatest threat in the mid-long term to this rare species seems to be landscape change resulting from the abandonment of traditional pastoral systems, which allows vegetation succession and reduces the area of suitable habitat [59,117].

Similarly, despite the influence of agroforestry management on habitat suitability of *Taxus baccata* at broader and coarser scales, climate (particularly temperature over precipitation) and productivity gained importance at more local and finer scales (see Table 5; Fig 5). In that sense, the most suitable areas to find this species could be characterized by non-cropped mountainous areas with cool summers, and cold and low productive winters, with a combination of open deciduous woodlands and tall scrub. *Taxus baccata* can form dense continuous patches in most of Europe, but in its southern limit (Mediterranean mountains), yew occurs as small patches or isolated trees in deciduous woods dominated by other species (HD habitat 9580* Mediterranean *Taxus baccata* woods), therefore contributing to forest mosaic diversity [60,62–64]. In the Iberian Peninsula, *Taxus baccata* thrives better in mixed and coniferous forests, mostly on limestone substrates and often occupying rocky cliffs and slopes [60]. However, on acid soils, the species competes worse under canopy and usually occurs in the form of small patches or isolated trees, as we observed in our focal National Park. Despite Mediterranean yew woods are protected under the HD, there are evident signs of regression in south-western Europe, mainly caused by changes in land-use and fire regimes [62].

### Spatial consistency of habitat suitability maps

To support the incorporation of EFA-based SDMs in reporting the conservation status of protected species (e.g. [118]), we also evaluated the spatial consistency of habitat suitability maps derived from models based on climatic and land-cover predictors and from models based on EFAs. The highest spatial consistency among maps of predicted habitat suitability was found between models based on climate plus land-cover and models based on EFAs, both for the narrowly and the widely distributed species. The pairwise comparisons between prediction maps showed that similarity was greater at more local and finer scales for the narrowly distributed species (see Table 6), while for the widely distributed species similarity was greater at larger and coarser scales (see Table 7).

Overall, projections from EFA-based models were more conservative in terms of predicted suitable area than those from models based on traditional predictors (see Figs 6 and 7). In addition, the spatial autocorrelation of predictions was lower in EFA-based models, probably due to the decoupling of ecosystem functioning from climate with increasing human land-use [119]. While climate factors usually describe more homogeneous and therefore connected areas at coarse scales, EFAs provided a response combining climate and landscape conditions at more local scales. EFA-based models even predict more fragmented areas, and therefore with less spatial correlation, than structural or compositional predictors [42,50]. This EFA-based response could be related to the dominant vegetation type, since areas covered by scrubs, which are characterized by high heterogeneity and diversity of species, show higher spatial variability and lower spatial autocorrelation than forest areas, usually more homogeneous in terms of species [119]. In this sense, the multi-scale analysis of the spatiotemporal dynamics of vegetation using satellite-derived EFAs could provide early-warnings of changes in habitat suitability beyond assessments based on climate interpolated surfaces or structural metrics such as those derived from land-cover maps [25].

In summary, our results confirm the spatial consistency of habitat suitability maps derived from EFAs-based models with those derived from models based on climate plus land-cover, confirming these annual descriptors of ecosystem functioning as useful SDM predictors for predicting and monitoring the habitat suitability for threatened species with different distribution ranges.

### Implications for assessment, monitoring and reporting

Complying with the legal obligations of EU member-states under Article 17^th^ of the Habitats Directive [53] requires providing extensive data on relevant parameters or indicators (e.g. range, number and dimension of populations, suitable habitat and future prospects) to report on the conservation status of species and habitats [118]. This assessment is often done at regional and coarse scales. However, for planning effective management actions on the ground, conservation priorities must be established at various scales [120–122]. Identifying local conservation priorities requires high-resolution data, not only concerning the distribution of species and habitats, but also documenting the environmental factors involved. However, these are often unavailable or are expensive to collect in situ. Most of these drawbacks can be mitigated thanks to remote sensing data, which have showed high potential for predicting and monitoring species distributions and habitat suitability at different scales [25,26,30].

Standard scenarios of biologically meaningful variables derived from remote sensing for projecting SDMs into the future, or for analyses of past dynamics, are currently still not available, but efforts are being developed by the scientific community towards the development of these products [123]. Several studies e.g. [35,50,110,116], and our results, have confirmed that remotely sensed variables related to ecosystem functioning (e.g. EFAs) can be useful predictors for modelling protected species distribution and habitat suitability. Satellite-derived EFAs hold the advantage of capturing similar environmental dynamics as traditional predictors (climate and landscape structure and composition), but incorporating further functional information [37,45]. Nevertheless, to capture early evidences of changes in vegetation dynamics that affect habitat suitability, analyses at different spatial and temporal levels of detail are required [122]. With our multi-scale modelling framework, tested on two contrasting species of conservation concern [53], we confirmed the potential of satellite-derived ecosystem functional variables as useful predictors for developing robust SDMs and monitoring habitat suitability predictions across scales. In this sense, multi-scale approaches (as implemented in this study), combined with long-term monitoring programs that incorporate high-spatial and temporal resolution data (e.g. Sentinel-2), may support broad scale habitat quality assessment for whole landscapes or regions, and at fine scales for individual protected areas. Since effective conservation of vulnerable and/or rare species requires an accurate identification of habitat suitability patterns and factors [109], we encourage the use of satellite-derived EFAs as a cost-effective source of standardized and repeatable measurements in upcoming biodiversity monitoring and reporting schemes. Moreover, our proposed multi-scale framework will be valuable for conservation managers to improve decision-making processes and for guiding conservation actions towards a more efficient and sustainable management.

In conclusion, we would highlight the following three major reasons supporting the use of Ecosystem Functional Attribute (EFAs) instead of (or besides) traditional climate and land cover variables: (1) EFAs are descriptors of ecosystem processes that may closely affect species distributions, while capturing other effects besides climate (e.g. those related to habitat features); (2) satellite-based predictors can nowadays be computed from imagery available at high frequencies and several spatial resolutions (to match the scale of the relevant processes in each study); and (3) EFA-based models have been shown to anticipate species’ responses to climate change scenarios. Due to these and other advantages, EFA-based models hold high potential for adaptive management of species, habitats and protected areas facing global change, even if they do not significantly outperform models based on traditional climate and land cover predictors under particular modelling setups.

## Supporting information

S1 Dataset *Iris boissieri* presence data at NW extent and 1km cell size, and joined values for each selected predictor to each occurrence data. (CSV)

S2 Dataset *Taxus baccata* presence data at NW extent and 1km cell size, and joined values for each selected predictor to each occurrence data. (CSV)

S1 Fig Correlation matrix for predictor variables used in model fitting in the Peneda-Gerês National Park (NP) extent and 1km cell size combinations for *Iris boissieri*. Legend. Spearman’s correlation matrix for all the variables used in model fitting (Note: the lowest absolute pairwise-correlation values were highlighted in green).
(TIF)

S2 Fig Correlation matrix for predictor variables used in model fitting in the Peneda-Gerês National Park (NP) extent and 1km cell size combinations for *Taxus baccata*. Legend. Spearman’s correlation matrix for all the variables used in model fitting (Note: the lowest absolute pairwise-correlation values were highlighted in green).
(TIF)

S3 Fig Correlation matrix for predictor variables used in model fitting in the North-western Iberian Peninsula (NW) extent and 1km cell size combinations for *Iris boissieri*. Legend. Spearman’s correlation matrix for all the variables used in model fitting (Note: the lowest absolute pairwise-correlation values were highlighted in green).
(TIF)

S4 Fig Correlation matrix for predictor variables used in model fitting in the North-western Iberian Peninsula (NW) extent and 1km cell size combinations for *Taxus baccata*. Legend. Spearman’s correlation matrix for all the variables used in model fitting (Note: the lowest absolute pairwise-correlation values were highlighted in green).
(TIF)

S5 Fig Multicollinearity test of predictor variables used in model fitting Peneda-Gerês National Park (NP-1km) for *Iris boissieri* (Ib) and *Taxus baccata* (Tb), by calculating the Variance Inflation Factors (VIF) as implemented in the R package *car* using function *vif()*. Legend. For each predictor, the VIF shows the collinearity degree among at least one independent variable with a combination of the other independent variables. In general, if VIF >5 is indicative of multicollinearity problems (Ringle et al., 2015).
(TIF)

S6 Fig Multicollinearity test of predictor variables used in model fitting North-western Iberian Peninsula (NW-1km) for *Iris boissieri* (Ib) and *Taxus baccata* (Tb), by calculating the Variance Inflation Factors (VIF) as implemented in the R package *car* using function *vif()*. Legend. For each predictor, the VIF shows the collinearity degree among at least one independent variable with a combination of the other independent variables. In general, if VIF >5 is indicative of multicollinearity problems (Ringle et al., 2015).
(TIF)

S7 Fig Model performance based on TSS (True Skills Statistics) for different extent (IP: Iberian Peninsula, NW: North-western IP and NP: Peneda-Gerês National Park) and spatial resolution (1km and 5km) combinations for *Iris boissieri* and *Taxus baccata*. Legend. Performance of the individual models (boxplots) showing the TSS_median_, two hinges (first and third quartiles), and two whiskers of each model filtered at TSS≥0.2 (empty-triangle signs represent the TSS_mean_). Filled-circle dots represent the TSS_median_ of the ensemble models.
(TIF)

S8 Fig Regression models among the median values of the Area Under the Curve (AUC) and the True Skills Statistics (TSS) of the ensemble models for *Iris boissieri* and *Taxus baccata*. Legend. Regression analysis among the AUC_median_ and TSS_median_ values for the ensemble models considering all extent (IP: Iberian Peninsula, NW: North-western IP and NP: Peneda-Gerês National Park) and spatial resolution (1km and 5km) combinations for *Iris boissieri* and *Taxus baccata*.
(TIF)

S9 Fig Variables importance for the best traditional (climate and land-cover)-based and satellite-derived Ecosystem Functional Attribute (EFA)-based models for *Iris boissieri*. Legend. Mean ± standard deviation variable importance of each predictor considered for the best-performed models (through all individual combinations of pseudoabsences, model runs and individual algorithms) fitted for *Iris boissieri* at all extents and spatial resolutions.
(TIF)

S10 Fig Variables importance for the best traditional (climate and land-cover)-based and satellite-derived Ecosystem Functional Attribute (EFA)-based models for *Taxus baccata*. Legend. Mean ± standard deviation variable importance of each predictor considered for the best-performed models (through all individual combinations of pseudoabsences, model runs and individual algorithms) fitted for *Taxus baccata* at all extents and spatial resolutions.
(TIF)

S11 Fig Spatial projections for *Iris boissieri*. Legend. Presence-absence maps for *Iris boissieri* from ensemble forecasting modelling calibrated by traditional predictors and satellite-derived ecosystem functional attributes (EFAs).
(TIF)

S12 Fig Spatial projections for *Taxus baccata*. Legend. Presence-absence maps for *Taxus baccata* from ensemble forecasting modelling calibrated by traditional predictors and satellite-derived ecosystem functional attributes (EFAs).
(TIF)
