# Supplementary figures and images for: Assessing the multi-scale predictive ability of ecosystem functional attributes for species distribution modelling
Source: PLoS One. 2018 Jun 18;13(6):e0199292. doi: 10.1371/journal.pone.0199292 (PMC6005496; doi:10.1371/journal.pone.0199292)

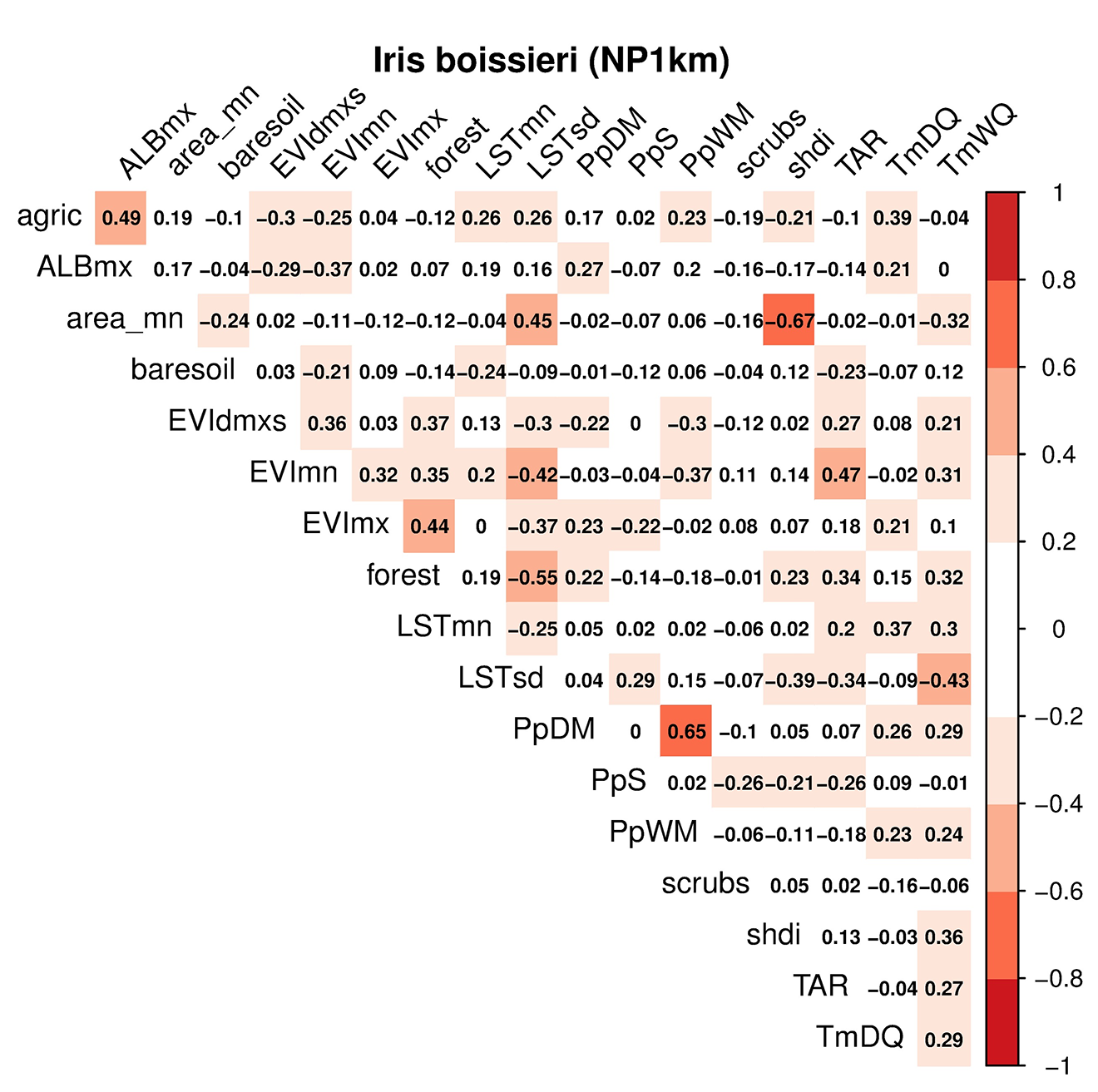

Supplement: S1 Fig — Legend. Spearman’s correlation matrix for all the variables used in model fitting (Note: the lowest absolute pairwise-correlation values were highlighted in green). (TIF) [file pone.0199292.s003.tif]

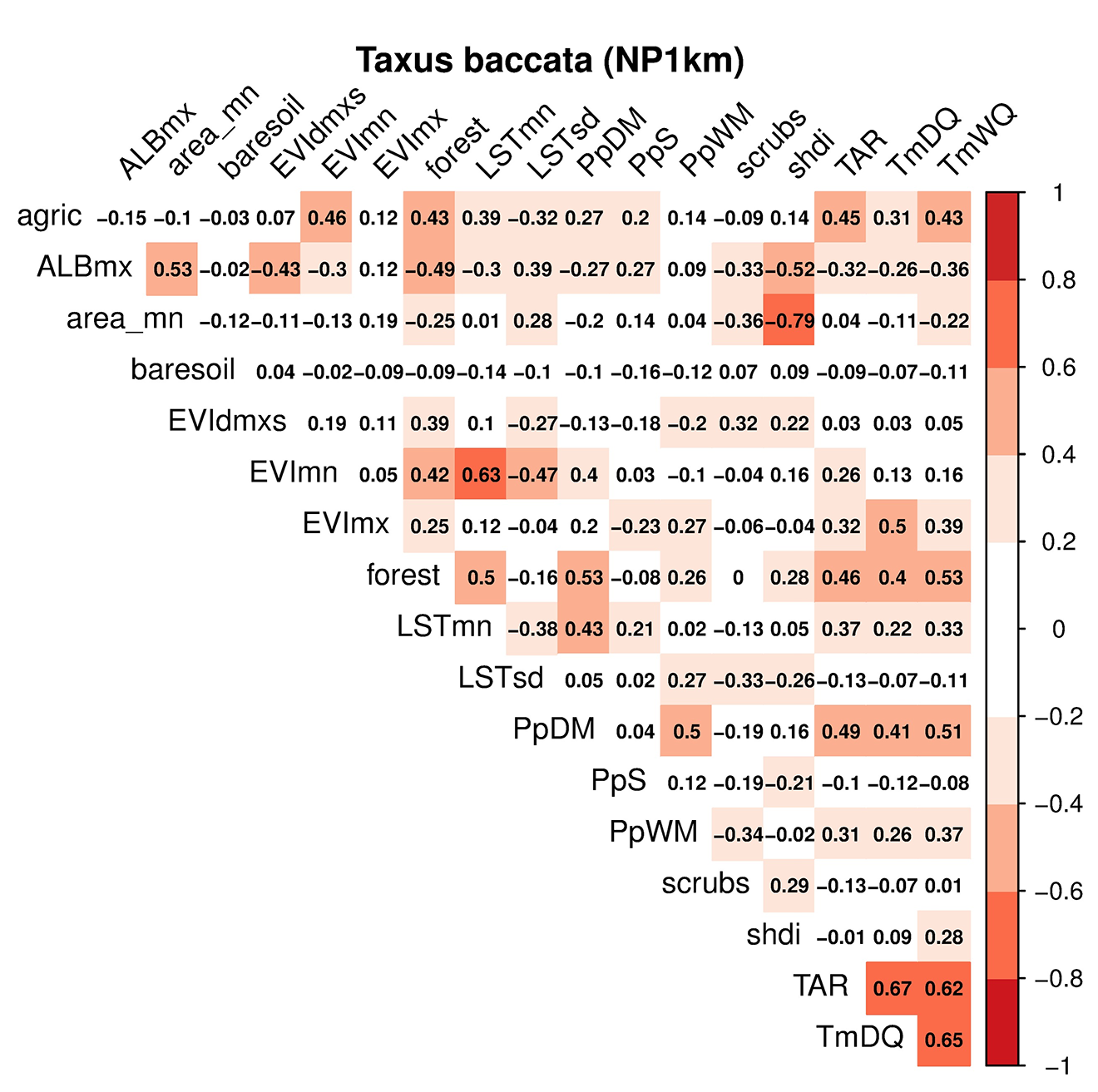

Supplement: S2 Fig — Legend. Spearman’s correlation matrix for all the variables used in model fitting (Note: the lowest absolute pairwise-correlation values were highlighted in green). (TIF) [file pone.0199292.s004.tif]

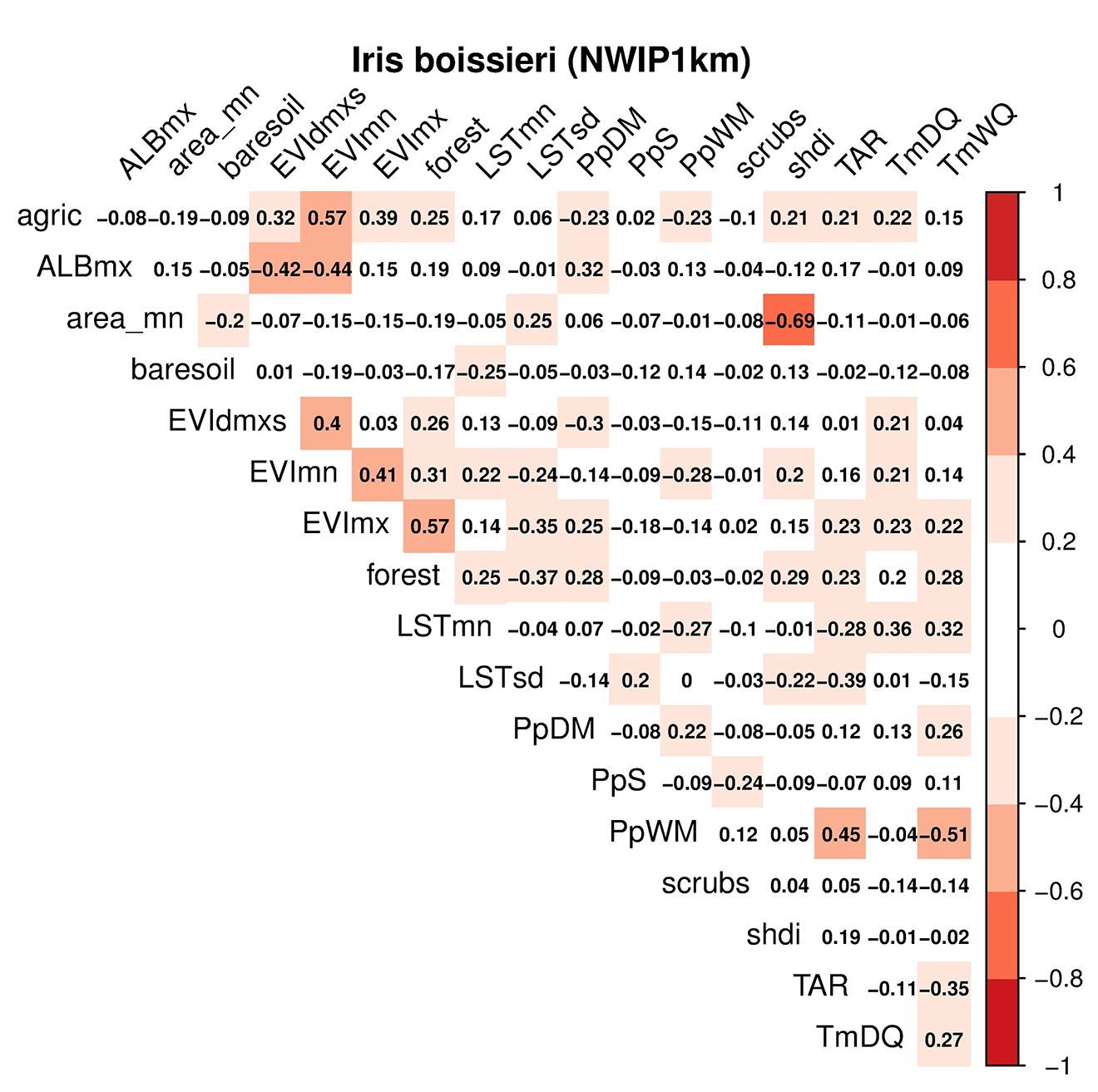

Supplement: S3 Fig — Legend. Spearman’s correlation matrix for all the variables used in model fitting (Note: the lowest absolute pairwise-correlation values were highlighted in green). (TIF) [file pone.0199292.s005.tif]

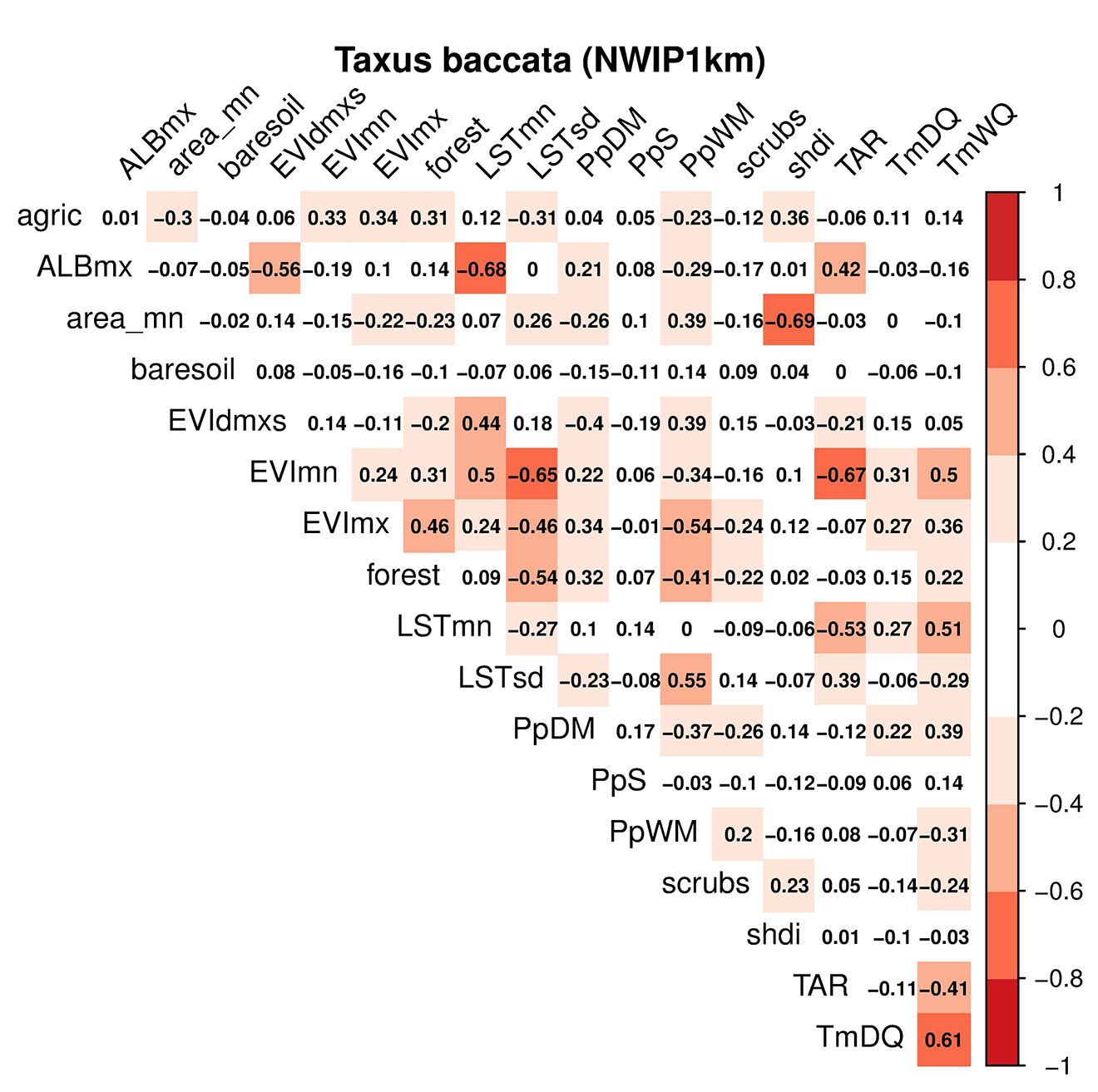

Supplement: S4 Fig — Legend. Spearman’s correlation matrix for all the variables used in model fitting (Note: the lowest absolute pairwise-correlation values were highlighted in green). (TIF) [file pone.0199292.s006.tif]

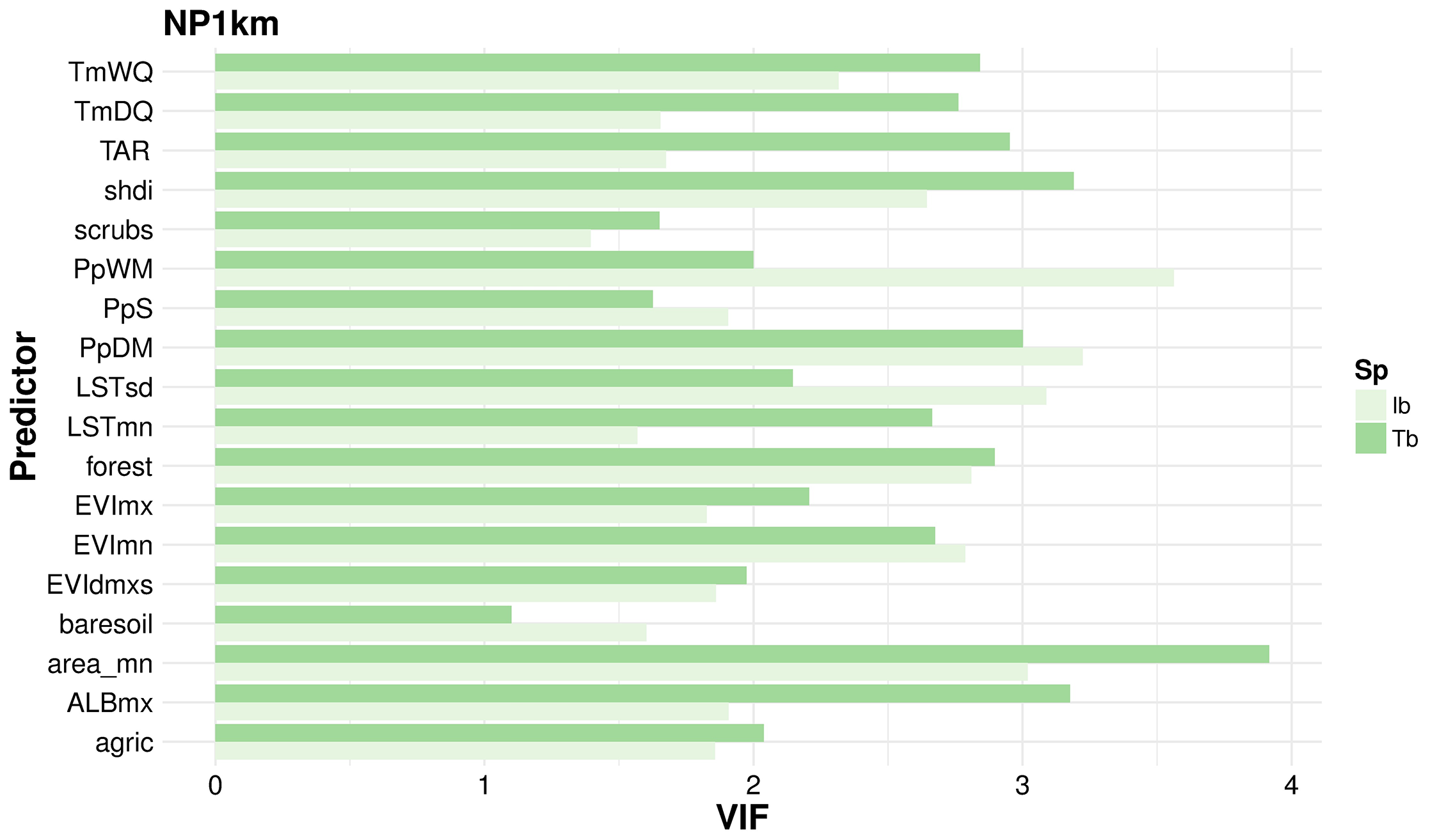

Supplement: S5 Fig — Legend. For each predictor, the VIF shows the collinearity degree among at least one independent variable with a combination of the other independent variables. In general, if VIF >5 is indicative of multicollinearity problems (Ringle et al., 2015). (TIF) [file pone.0199292.s007.tif]

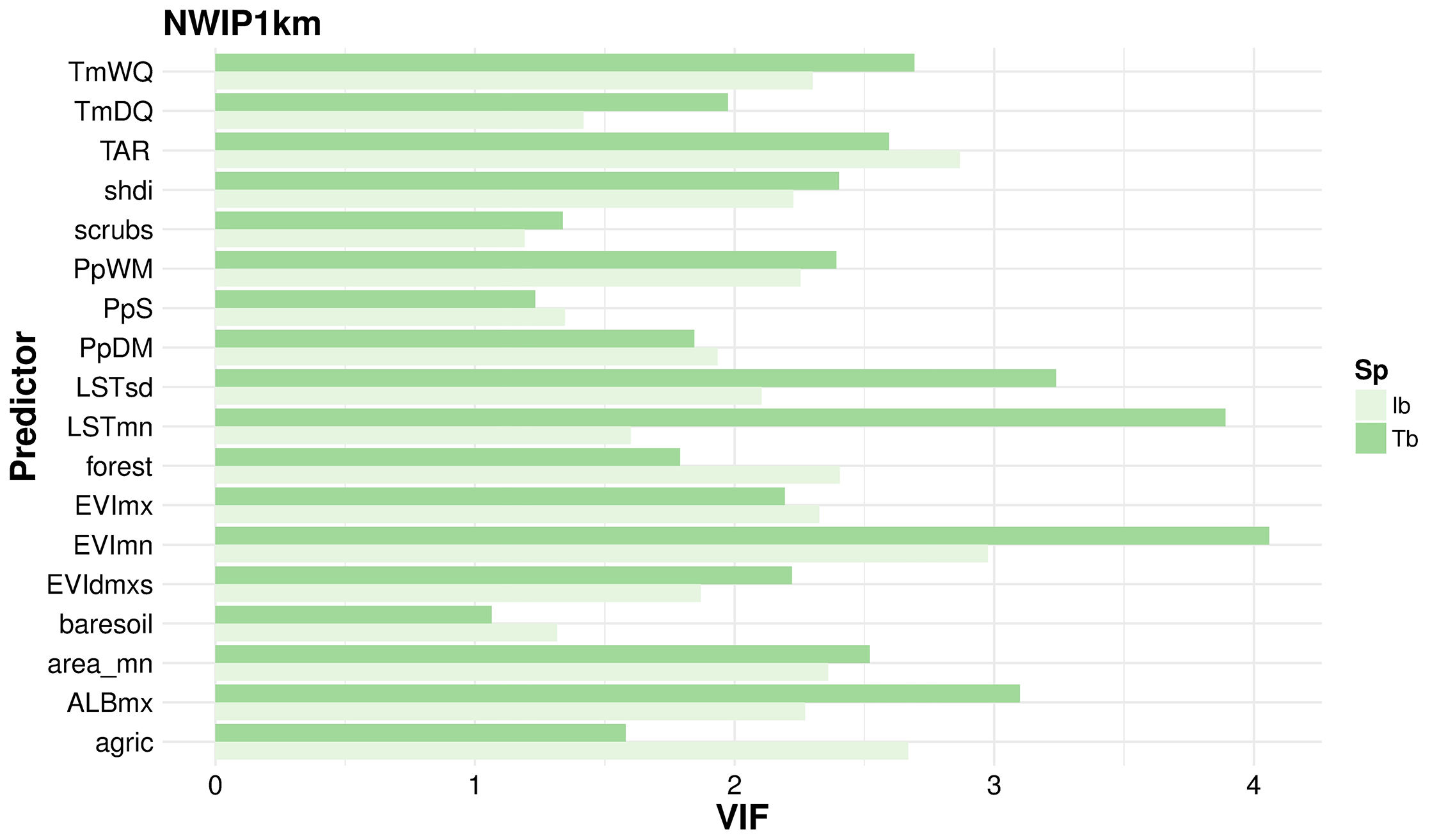

Supplement: S6 Fig — Legend. For each predictor, the VIF shows the collinearity degree among at least one independent variable with a combination of the other independent variables. In general, if VIF >5 is indicative of multicollinearity problems (Ringle et al., 2015). (TIF) [file pone.0199292.s008.tif]

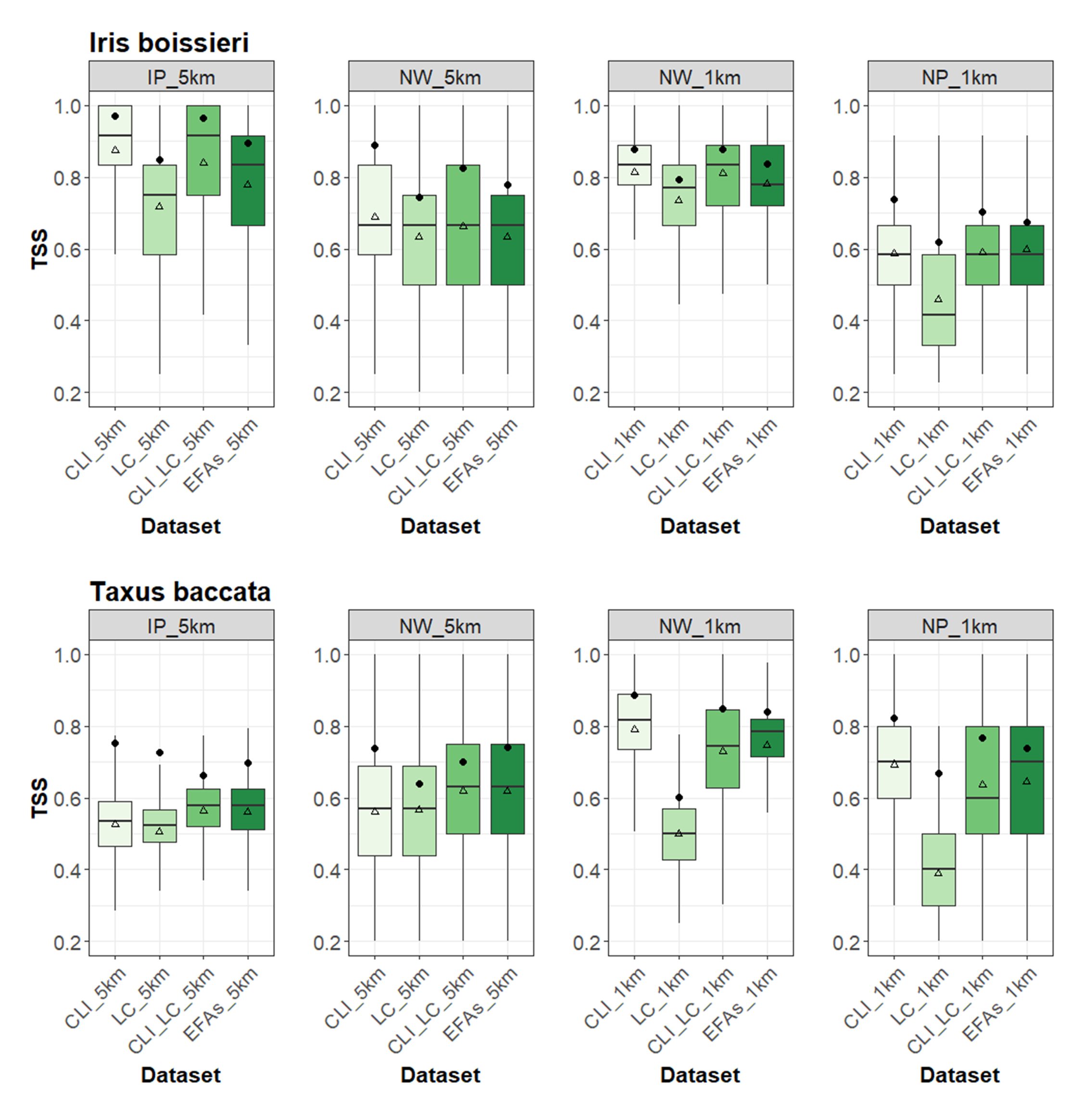

Supplement: S7 Fig — Legend. Performance of the individual models (boxplots) showing the TSSmedian, two hinges (first and third quartiles), and two whiskers of each model filtered at TSS≥0.2 (empty-triangle signs represent the TSSmean). Filled-circle dots represent the TSSmedian of the ensemble models. (TIF) [file pone.0199292.s009.tif]

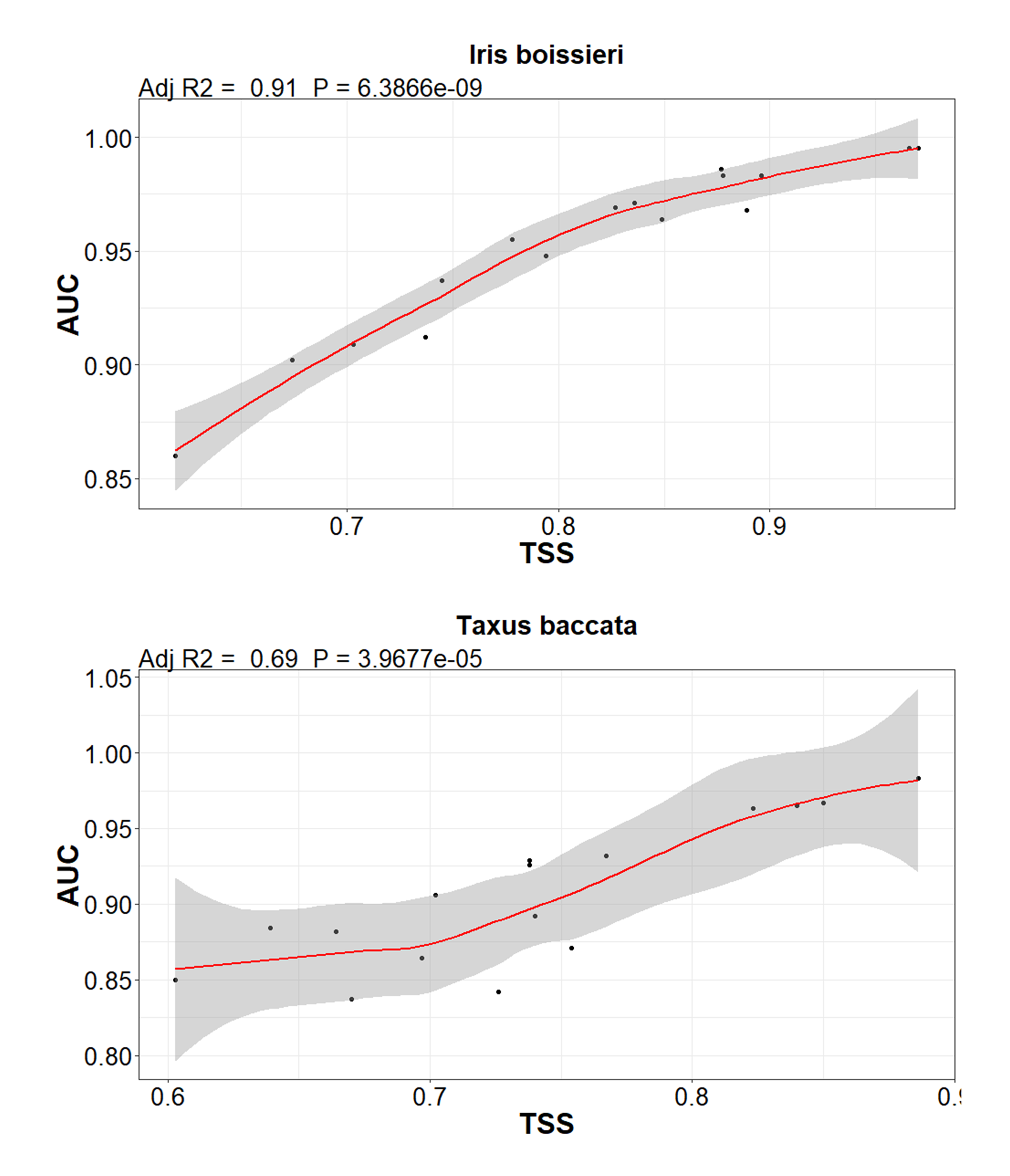

Supplement: S8 Fig — Legend. Regression analysis among the AUCmedian and TSSmedian values for the ensemble models considering all extent (IP: Iberian Peninsula, NW: North-western IP and NP: Peneda-Gerês National Park) and spatial resolution (1km and 5km) combinations for Iris boissieri and Taxus baccata. (TIF) [file pone.0199292.s010.tif]

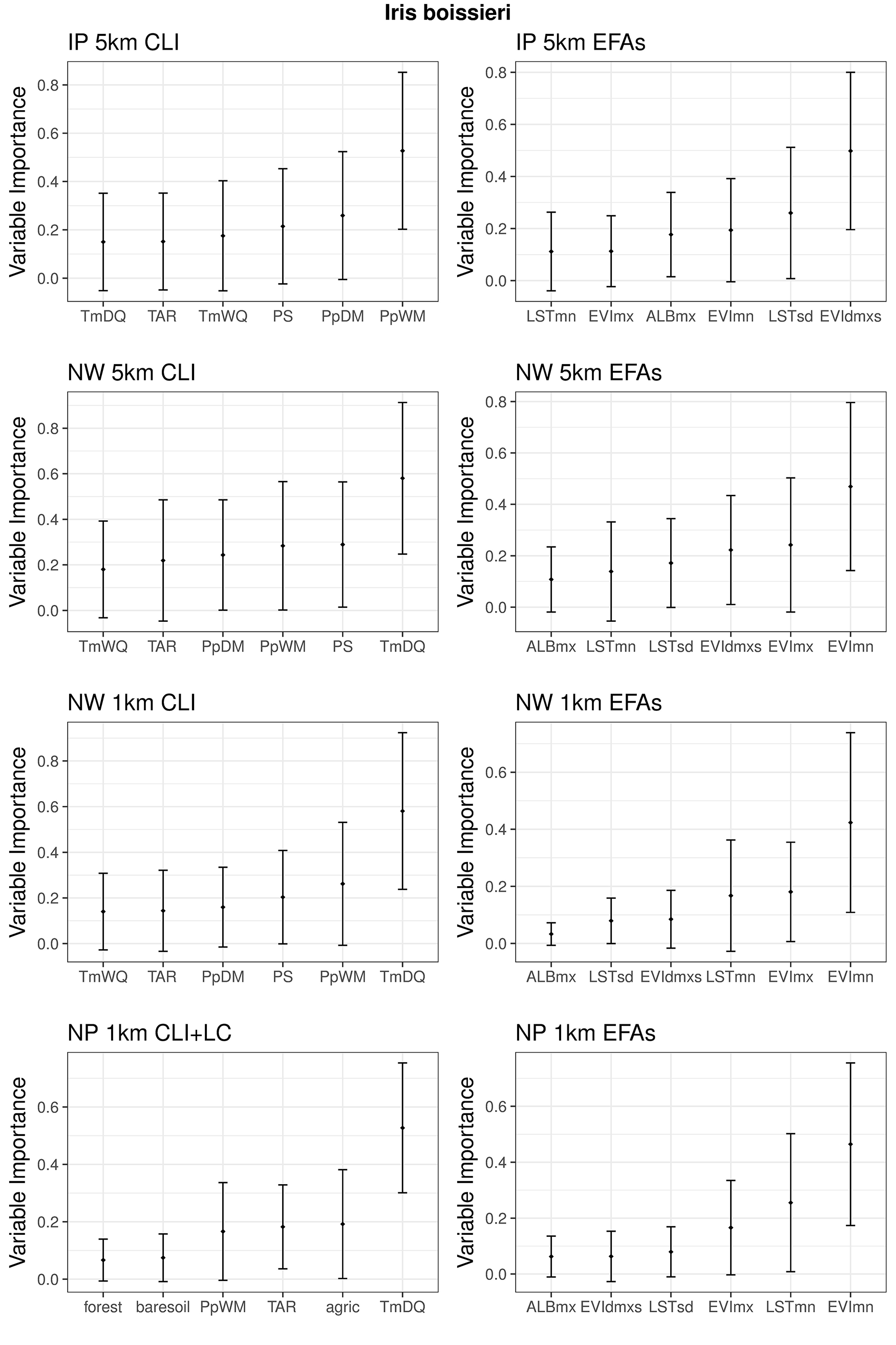

Supplement: S9 Fig — Legend. Mean ± standard deviation variable importance of each predictor considered for the best-performed models (through all individual combinations of pseudoabsences, model runs and individual algorithms) fitted for Iris boissieri at all extents and spatial resolutions. (TIF) [file pone.0199292.s011.tif]

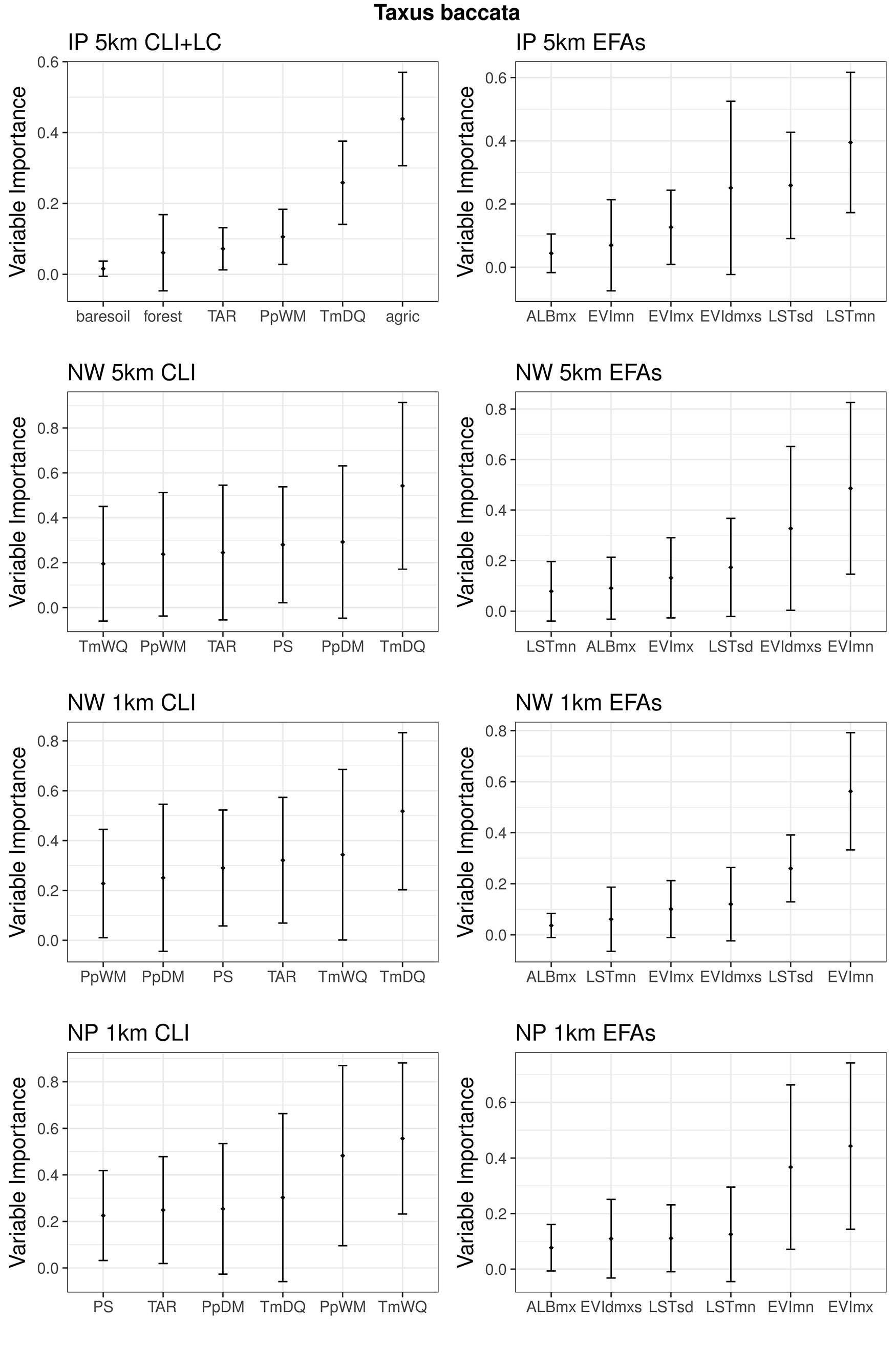

Supplement: S10 Fig — Legend. Mean ± standard deviation variable importance of each predictor considered for the best-performed models (through all individual combinations of pseudoabsences, model runs and individual algorithms) fitted for Taxus baccata at all extents and spatial resolutions. (TIF) [file pone.0199292.s012.tif]

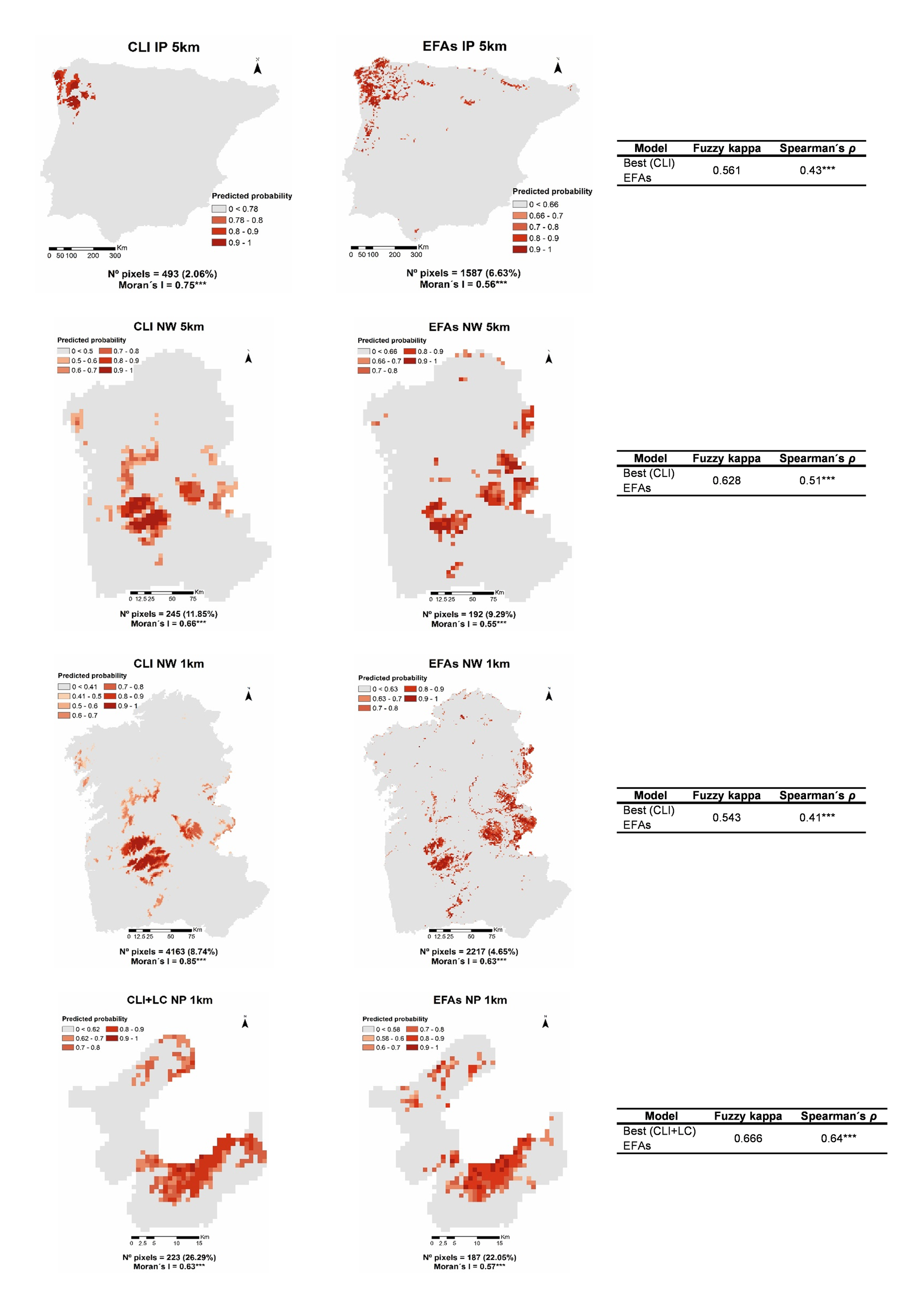

Supplement: S11 Fig — Legend. Presence-absence maps for Iris boissieri from ensemble forecasting modelling calibrated by traditional predictors and satellite-derived ecosystem functional attributes (EFAs). (TIF) [file pone.0199292.s013.tif]

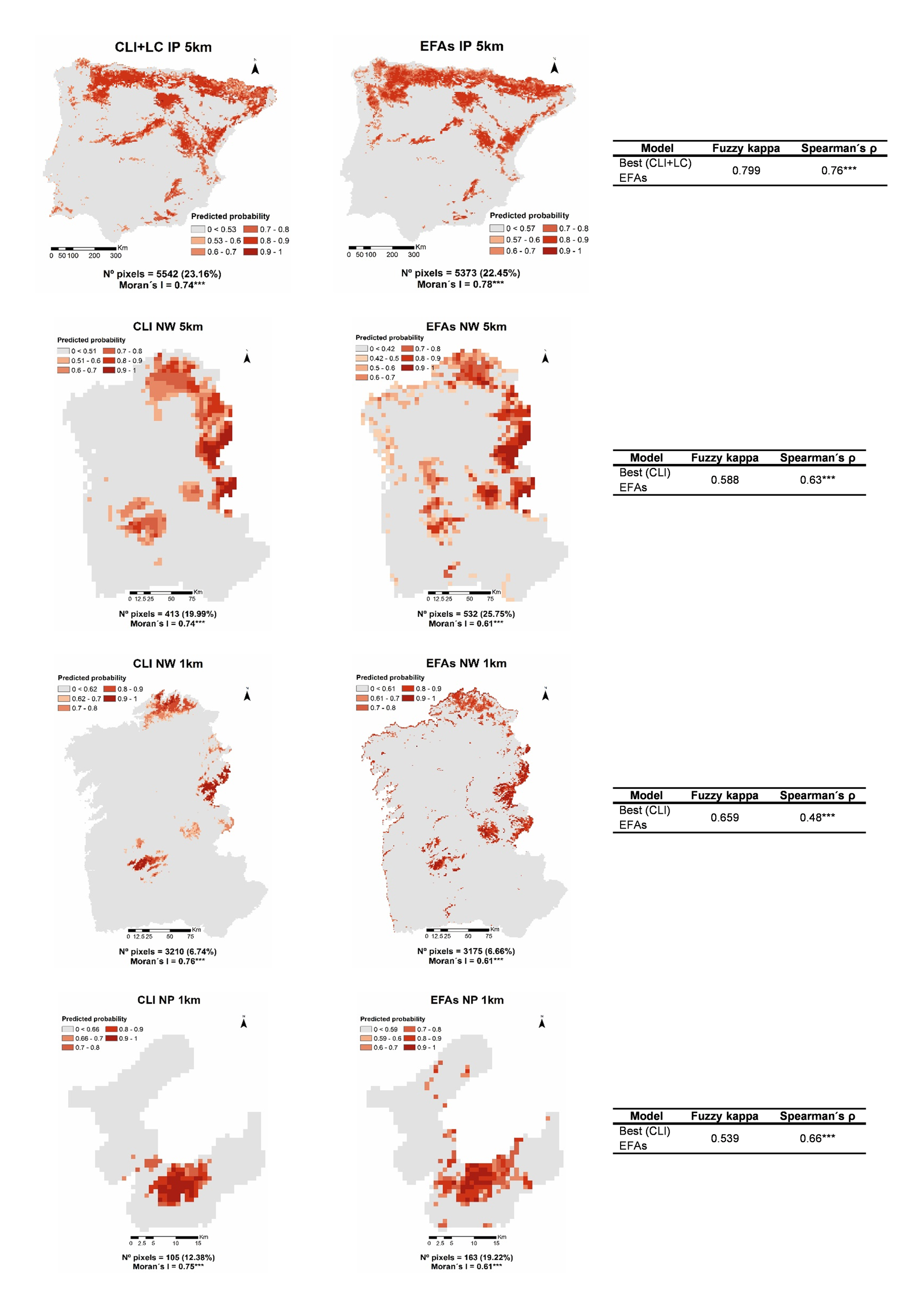

Supplement: S12 Fig — Legend. Presence-absence maps for Taxus baccata from ensemble forecasting modelling calibrated by traditional predictors and satellite-derived ecosystem functional attributes (EFAs). (TIF) [file pone.0199292.s014.tif]
